# A Systematic and Comparative Review of Behavior Change Strategies in Stress Management Apps: Opportunities for Improvement

**DOI:** 10.3389/fpubh.2022.777567

**Published:** 2022-02-24

**Authors:** Mona Alhasani, Dinesh Mulchandani, Oladapo Oyebode, Nilufar Baghaei, Rita Orji

**Affiliations:** ^1^Faculty of Computer Science, Dalhousie University, Halifax, NS, Canada; ^2^Games and Extended Reality Lab, Massey University, Auckland, New Zealand

**Keywords:** stress management, persuasive technology, persuasive strategies, systematic review, design recommendations, mobile health apps, mental health

## Abstract

Stress is one of the significant triggers of several physiological and psychological illnesses. Mobile health apps have been used to deliver various stress management interventions and coping strategies over the years. However, little work exists on persuasive strategies employed in stress management apps to promote behavior change. To address this gap, we review 150 stress management apps on both Google Play and Apple's App Store in three stages. First, we deconstruct and compare the persuasive/behavior change strategies operationalized in the apps using the Persuasive Systems Design (PSD) framework and Cialdini's Principles of Persuasion. Our results show that the most frequently employed strategies are *personalization*, followed by *self-monitoring*, and *trustworthiness*, while social support strategies such as *competition, cooperation* and *social comparison* are the least employed. Second, we compare our findings within the stress management domain with those from other mental health domains to uncover further insights. Finally, we reflect on our findings and offer eight design recommendations to improve the effectiveness of stress management apps and foster future research.

## Introduction

Stress is a common and continuous phenomenon that poses mild to serious physical and mental health risks to individuals (1). Stress is in various forms such as routine stress (i.e., pressures from work, family, school, and other daily activities), traumatic stress (caused by events such as accidents, war, harassment or assault, natural disasters, disease outbreak, etc.), and others such as stress due to sudden negative events like divorce, sickness, and job loss (2). Stress can be short-term (acute stress) or repeatedly occur over a long term (chronic stress). Evidence shows that more than three-quarters of adults in the United States report physical or emotional stress symptoms including headache, tiredness, and sleep disorders in 2019 (3). Moreover, stress has been linked to coronary heart disease (4), Type 2 diabetes (5), cardiovascular disease (6–9), childhood asthma, and wheezing due to maternal stress during pregnancy (10), osteoporosis (11), miscarriage (12), menstrual problems (13), acne (14), obesity (15), autoimmune disease (16), and Alzheimer's disease (17). The mental health consequences of stress (e.g., anxiety, depression, panic attacks, sleep disorders, and low cognitive performance) have also been documented (18–22). In addition, maternal stress during pregnancy has been shown to negatively affect offspring's neuro and cognitive development, triggering negative affectivity, difficult temperament, and psychiatric disorders (23). Stress can be reduced or managed through mindfulness and meditation (24–32), music (33), and nature relatedness (34, 35), cognitive-behavioral therapy (36–38), stress-reduction programs (31, 39), and structured counseling (40, 41).

In recent years, stress management interventions have been delivered to target audiences through mobile health (mHealth) apps due to the proliferation of smartphones (42) and advancements in smartphone technology. These apps are capable of detecting stress through continuous monitoring of heart rate variability (43, 44) using smartphone sensors (e.g., photoplethysmography) or wearable devices such as heart-rate chest strap and electrocardiography (45). In addition, mHealth apps can detect stress through skin conductance (46) or self-reports (such as daily mood logs) (47). Furthermore, mHealth apps can deliver real-time stress relief or relaxation features (such as breathing sessions, mindfulness and meditation practice, emotion regulation programs, etc.) using biofeedback techniques (48–50), conversational coaches (51), gamification, videos, etc. Evidence shows people's willingness to adopt mHealth apps for their stress relief needs (52). Research also indicates that mHealth apps are effective in reducing stress and improving users' wellbeing over time (53, 54). To promote continuous engagement with stress management apps, designers/developers operationalized various persuasive or behavior change strategies, whether consciously or unconsciously, to help users deal with their stress. Although systematic reviews of persuasive health apps exist in the literature (55–57), little is known about the persuasive strategies employed in stress management apps to motivate behavior change, to the best of our knowledge.

In this paper, we reviewed and analyzed 150 stress management apps on both Google Play and App Store with the aim of deconstructing the persuasive and behavior change techniques they employed using the Persuasive Systems Design (PSD) framework (58) and Cialdini's Principles of Persuasion (CPP) (59) which are two widely employed frameworks in persuasive and behavior change systems design. Specifically, we address the following research questions:

**RQ1:** What are the most and least employed strategies in stress management apps?
**RQ2:** How are the strategies operationalized or implemented in these apps?
**RQ3:** How do stress management apps compare with apps in other mental health domains in terms of the persuasive strategies employed?

## Background

### Persuasive Strategies and Persuasion Frameworks

Fogg et al. defined persuasive technology as “interactive computing systems designed to change people's attitudes and behaviors” (60). Similarly, Oinas-Kukkonen et al. defined persuasive systems as “computerized software or information systems designed to reinforce, change or shape attitudes or behaviors or both without using coercion or deception” (61). Both Fogg and Oinas-Kukkonen proposed persuasion frameworks that are useful in analyzing and designing persuasive technologies—the Fogg Behavior Model (FBM) (62) and Persuasive Systems Design (PSD) framework (58), respectively. The FBM posits that a person must meet three conditions to perform a target behavior: (i) be sufficiently motivated, (ii) possess the ability to perform the behavior, (iii) receive the trigger to perform the behavior. Hence, behavior is a combination of three factors that must occur at the same time: motivation, ability, and triggers. The PSD framework builds upon the FBM to describe how to inject persuasive features that motivate behavior change into systems during the design process. The PSD framework comprises 28 persuasive strategies grouped into four categories—*primary task support, dialogue support, system credibility support*, and *social support*. The primary task support category comprises persuasive strategies that support users in carrying out their primary task. In contrast, the dialogue support category includes persuasive strategies that provide some degree of system feedback to users. The system credibility support category comprises persuasive strategies that support designing a system that it is more credible and thus more persuasive. The social support category includes persuasive strategies that enable system users to interact or socialize with others. Table 1 shows the four categories and their respective persuasive strategies, as well as a description of each strategy according to (58).

**Table 1:** Twenty-eight persuasive strategies in the Persuasive Systems Design (PSD) framework.

**Persuasive strategy**	**Description**
**Primary task support**	
Reduction	Reduces users' effort by breaking complex behaviors into simple to help them perform the target behavior.
Tunneling	Guide users through a process to provide opportunities to encourage them along the way.
Tailoring	Provide Information will be more persuasive if it is tailored to the potential needs, interests, personality, usage context, or other factors related to a particular user group.
Personalization	Offer personalized content or customized services for users.
Self-monitoring	Allow users to track and monitor their performance, progress, or status in achieving their goals.
Simulation	Enable users to observe the link between the cause and effect of their behaviors.
Rehearsal	Provide means for users to rehearse their target behavior.
**Dialogue support**	
Praise	Offer praise through symbols, words, images or sounds as feedback for users to encourage their progress toward the target behavior.
Rewards	Provide virtual rewards for users when completing their target behaviors.
Reminders	Remind users of their target behavior to assist achieve their goals.
Suggestion	Provide appropriate suggestions for users to achieve their target behaviors.
Similarity	Remind users of themselves or adopt trending features in a meaningful way
Liking	Contain a visually attractive look and feel which meets users' desires.
Social role	Adopts a social role such as provide communication between users and the system's specialists.
**System credibility support**	
Trustworthiness	Provide truthful, reasonable, and unbiased information for users.
Expertise	Provide information showing competence, experience, and knowledge.
Surface credibility	Contain a competent look and feel that promot system credibility based on users' initial assessments.
Real-world feel	Show information about people or organizations behind the content or services.
Authority	Refer to people in the role of authority.
Third-party endorsements	Highlight endorsements from respected and well-known sources.
Verifiability	Provide means to investigate the accuracy of the content via external sources.
**Social support**	
Social learning	Allow users to observe other users' performance and outcomes while they are doing the same target behavior.
Social comparison	Allow users to compare their performances with other users.
Normative influence	Allow users to gather with other individuals who share the same objectives to feel norms.
Social facilitation	Enable users to discern other users who perform the target behavior
Cooperation	Motivate users to cooperate with other users to achieve the target behavior goal.
Competition	Motivate users to compete with other users to achieve the target behavior goal.
Recognition	Provide public recognition, such as ranking feature, for users who perform their target behavior.

Additional frameworks have emerged over the years, such as Cialdini's Principles of Persuasion (CPP) (59). CPP comprises six (6) persuasive strategies described below:

- ***Reciprocity*:** give gifts or freebies to users, and they will repay in kind
- ***Social Proof*:** use peer power whenever it is available
- ***Liking*:** uncover real similarities and offer genuine praise
- ***Consistency/commitment*:** make user commitments active, public, and voluntary
- ***Authority*:** demonstrate expertise, and
- ***Scarcity*:** highlight unique benefits and exclusive information.

Although the PSD framework is comprehensive and widely used to extract persuasive strategies implemented in mobile apps, the framework did not capture strategies such as *reciprocity, consistency/commitment*, and *scarcity* which are commonly implemented in apps for behavior change (57). In addition, *social proof, liking, and authority* strategies in CPP are similar to *social facilitation, liking*, and *authority* strategies in PSD. Hence, in this study, we focused on *reciprocity, consistency/commitment*, and *scarcity* from CPP in addition to the PSD strategies.

### Persuasive Strategies Employed in Mobile Health (mHealth) Apps

Previous research has investigated the persuasiveness of apps targeting various domains, including health, using the PSD framework only or in conjunction with other persuasion frameworks.

In the general area of health and wellness, Langrial et al. (63) reviewed 12 healthcare apps to identify the PSD framework's persuasive strategies. Their findings revealed that strategies in the primary task support category (especially *self-monitoring, reduction*, and *personalization*) were relatively broadly utilized, while dialogue support strategies were largely underutilized. In addition, the implementation of social support strategies was found to be shallow. Similarly, Oyebode et al. (57) conducted a comparative analysis of 80 health and wellness apps to evaluate the persuasive features implemented using the PSD and CPP frameworks. Their findings revealed that *personalization, self-monitoring, suggestion, trustworthiness, surface credibility*, and *liking* are the dominant persuasive strategies under the PSD framework, while *consistency/commitment* is the dominant strategy under CPP. In addition, persuasive strategies under the social support category were generally underutilized. To investigate the effectiveness of persuasive technologies for health and wellness, Orji and Moffatt (64) conducted a literature search and found that persuasive technologies effectively promote healthy behaviors, with 92% of reviewed articles reporting positive outcomes. Similarly, Hamari et al. (65) conducted empirical research on persuasive technologies (95 articles), and the results show that 92.6% of the reviewed articles reported positive outcomes.

In the area of mental health, Alqahtani et al. (55) analyzed 103 mental health apps to determine their persuasiveness using the PSD framework and Behavior Change Techniques (BCTs). Their results revealed that *self-monitoring* is the most frequently employed persuasive strategy, followed by *personalization, reminder*, and *rewards*. Similarly, Chang et al. (66) analyzed 12 mental health apps using the PSD framework and Goal-setting theory. Their findings showed that persuasive strategies in the primary task support categories, including goal setting, are the most employed, while social support strategies were the least employed.

In the domain of disease management, Geuens et al. (67) reviewed 28 mobile apps for managing chronic arthritis using the PSD framework and BCTs. Their results revealed that persuasive strategies in the system credibility support are the most employed, followed by strategies in the primary task support, dialogue support, and social support categories in decreasing order of utility. Similarly, Fadhil and Wang (68) investigated the persuasiveness of 19 diabetes management apps and coded them using the PSD framework and BCTs. Based on their findings, persuasive strategies in the primary task support category (especially *personalization, self-monitoring*, and *tailoring*) and system credibility support (especially *expertise* and *trustworthiness*) are the most employed. In contrast, dialogue support and social support strategies are the least employed.

Furthermore, Win et al. (69) examined the persuasive features employed in medication management apps retrieved from 13 research articles using the PSD framework. Based on their findings, persuasive strategies in the primary task support and dialogue support categories (such as *tailoring, self-monitoring*, and *reminders*) were widely employed in the apps, while those in the social support category were the least utilized. Furthermore, Lehto et al. applied the PSD framework to identify and analyze persuasive strategies in web-based alcohol and smoking interventions retrieved from 23 articles. Their findings showed that primary task support strategies (especially *reduction, self-monitoring, simulation*, and *personalization*) were relatively widely utilized, while persuasive strategies in the social support category were moderately utilized. In addition, dialogue support strategies were generally found to be underutilized.

In the physical activity domain, Matthews et al. (70) reviewed fitness apps from 20 research articles using the PSD framework. Their findings revealed that self-monitoring is the most widely represented strategy in the primary task support category, followed by *personalization* and *tunneling*, while suggestion is the most common dialogue support persuasive strategy. Almutari and Orji (71) focused on identifying social support strategies employed by physical activity apps in 32 studies using the PSD framework. They found that majority of the apps employed the *competition* persuasive strategy to promote social interaction, followed by the *cooperation* and *social comparison* strategies.

In summary, the literature review above shows that the dominance of the strategy utilization in persuasive and behavior change app design varies; however, there is no analysis targeting the stress management domain and how the results compare with other areas of mental health. Therefore, this paper utilized the PSD and CPP frameworks to deconstruct the persuasive strategies implemented in 150 stress management apps. We conducted a comparative analysis to understand the persuasive strategies employed in the apps and uncover new insights that drive future research.

## Methods

In this section, we discuss the app selection criteria and coding process.

### Selection of Stress Management Apps

**Figure 1:**
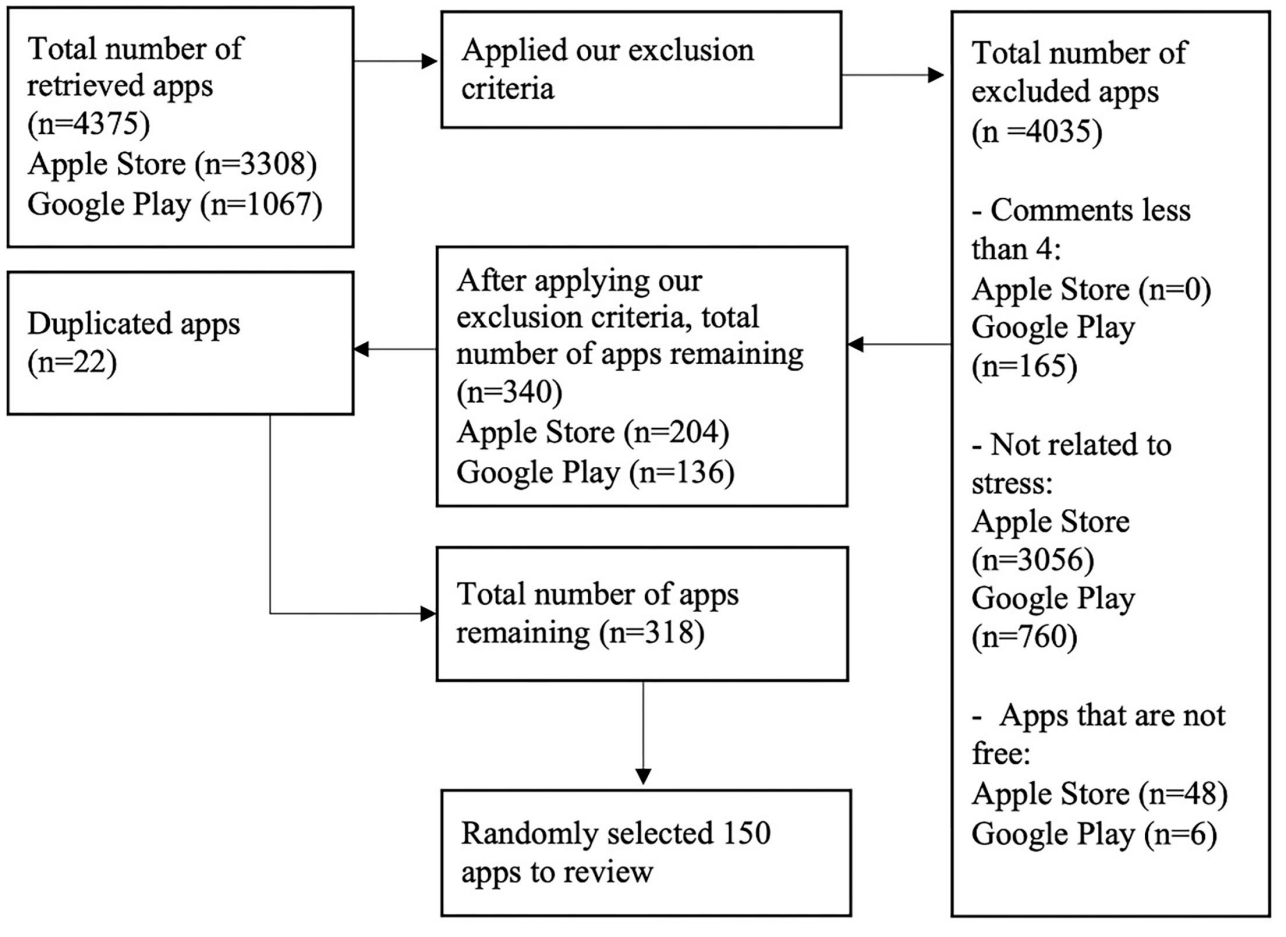
App selection process (150 apps selected).

Apps were extracted from Google Play and Apple's App Store automatically using a script that we developed using JavaScript and Node.js runtime. Search keywords were formulated by combining the term “stress” with each of the following terms: *management, monitoring, support, detection, mindfulness, meditation, breathing, relief*, *relaxation, curbing, prevention*, and *treatment*. The search was conducted in February 2020 and produced 4,375 apps in total (3,308 apps from App store and 1,067 from Google Play). For analysis, the inclusion criteria were: (1) apps must have more than four user comments, (2) apps must be related to stress, and (3) apps must be free or free with in-app purchases (free/free^*^). The apps that did not meet all of these criteria were excluded. To avoid duplication, any app that appeared on both platforms were considered as one. Information like app name, platform (i.e., iOS, Android, or both), developer name, free/free^*^ were collected. We randomly selected 150 out of the 318 eligible apps for review (see Figure 1). One hundred fifty is a good number for generalizable results.

### Process of Coding Apps for Persuasive Strategies

**Figure 2:**
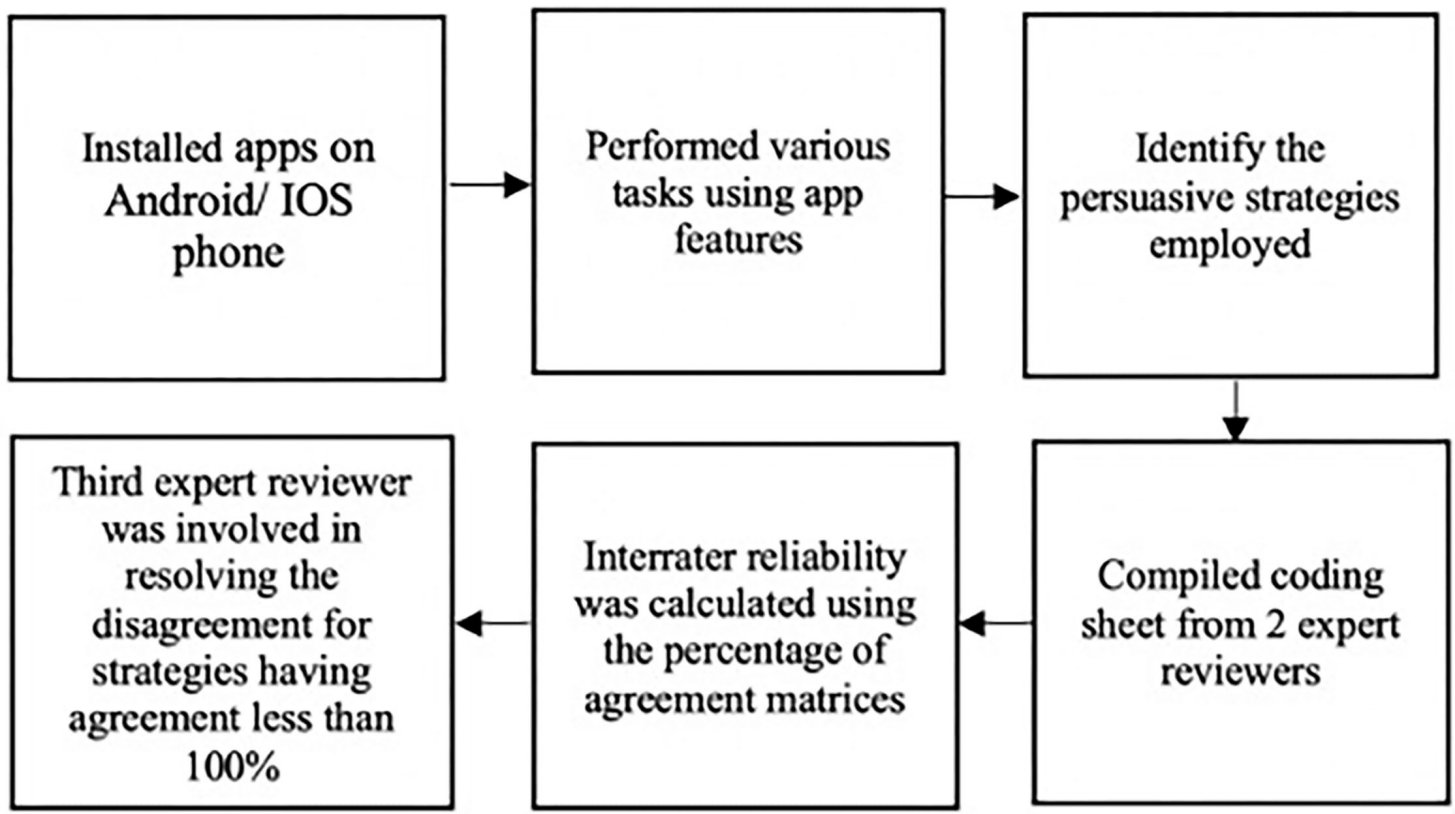
Process of coding apps.

The purpose of coding the apps in our research is to evaluate the number and type of persuasive strategies employed in mental health apps specifically related to stress. We collected 150 apps and deconstructed the persuasive strategies employed using the PSD and CPP frameworks. We combined both frameworks to boost the coverage of our coding process. Two persuasive technology researchers coded the apps after installing them and performing various tasks using the apps' features. For apps with in-app purchases, researchers accepted the free trial to enable examination of all persuasive strategies employed in the apps. The interrater agreement score for each strategy was computed after coding. Finally, a third expert reviewer was involved in resolving any disagreement for strategies having agreement score <100%. Figure 2 shows the steps of coding the apps. Appendices A, B present the summary of the apps evaluated and the persuasive strategies employed in the apps.

### Agreement

The interrater reliability for the coded apps was measured using the percentage of agreement metric (6), as shown in Table 2. Agreement occurs when both researchers indicate the presence or absence of a persuasive strategy in an app. Disagreement occurs if one reviewer indicates the presence of a strategy and the second reviewer indicates an absence. Reliability values range between 85.7 and 100% agreement depending on the persuasive strategy. The strategy with the lowest interrater reliability (85.7%) is social role, while 27 out of the 31 strategies obtained perfect agreement scores. Overall, all intercoder reliability scores are within the acceptable range [i.e., > 60% (72)].

**Table 2:** Agreement percentage of persuasive strategies.

**Persuasive strategies**	**Percentages of agreement**
**Primary task support**	
Personalization	98.4
Self-monitoring	100
Simulation	88.4
Tailoring	100
Rehearsal	100
Tunneling	100
Reduction	100
**Dialogue support**	
Reminders	100
Rewards	100
Suggestions	100
Praise	95.7
Social role	85.7
Liking	100
Similarity	100
**System credibility support**	
Trustworthiness	100
Real-world feel	100
Expertise	100
Authority	100
Verifiability	100
Surface credibility	100
Third-party endorsements	100
**Social support**	
Social learning	100
Social facilitation	100
Normative influence	100
Competition	100
Recognition	100
Social comparison	100
Co-operation	100
**Cialdini's principles of persuasion**	
Reciprocity	100
Commitment/Consistency	100
Scarcity	100

### Categories of Stress Management Apps Based on Their Purpose

**Figure 3:**
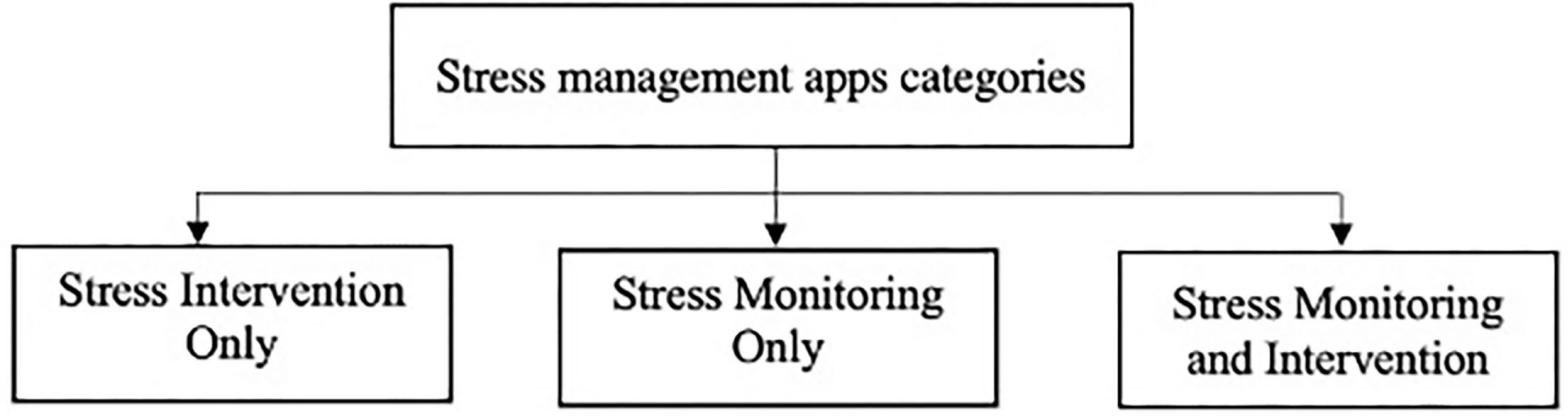
Stress management apps categorized according to their purpose.

We classified apps into three categories, as shown in Figure 3, based on the purpose intended by the app. Most apps were designed to provide different sets of interventions to help users relieve and cope with stress. For example, apps provide different relaxation sessions or a list of games to help reduce stress levels. However, some apps were developed to monitor or track users' stress levels by allowing them to record their feelings either manually or by capturing their stress in real-time via sensors. Few apps combined both stress monitoring (tracking) and intervention by detecting users' stress levels and providing interventions such as meditation and relaxation sessions.

## Results

This section describes the results of our analysis, including information about the apps reviewed, implementation of the persuasive strategies employed, and comparison between the different categories of persuasive strategies.

### Information on Selected Apps

Table 3 presents a summary of the reviewed apps.

**Table 3:** Summary of the selected stress management apps.

**Parameters**	**Description**	**Summary**
Price	Whether apps were totally free, or users need to make in-app purchases to access advanced features	Free (55%), Free with in-app purchases (45%)
Rating	Rating of apps in App Stores and Google Play Store	No rating (13%), 0–2.9 (2%), 3–3.9 (27%), 4–4.9 (52%), 5 (6%)
Platform	Whether apps were available in App Store or Google Play Store or both	iPhone (61%), Android (11%), Both (29%)
Target Issue	Target mental health issues that apps targeted	Stress only (74%), Stress and Anxiety (19%), Stress, Anxiety, Depression (5%), Stress, Anxiety, and Worry (1%), Stress and General Well-being (2%).
Last update	The last date apps were updated	2013–2014 (1%), 2015–2016 (13%), 2017–2018 (29%), 2019–2020 (57%)

### Persuasive Strategies and Their Implementation

To answer our second research question (RQ2), we identified how each persuasive strategy is operationalized or implemented in all 150 stress management apps. We discuss these implementations in the following subsections.

#### Primary Task Support Strategies and Their Implementation

**Figure 4:**
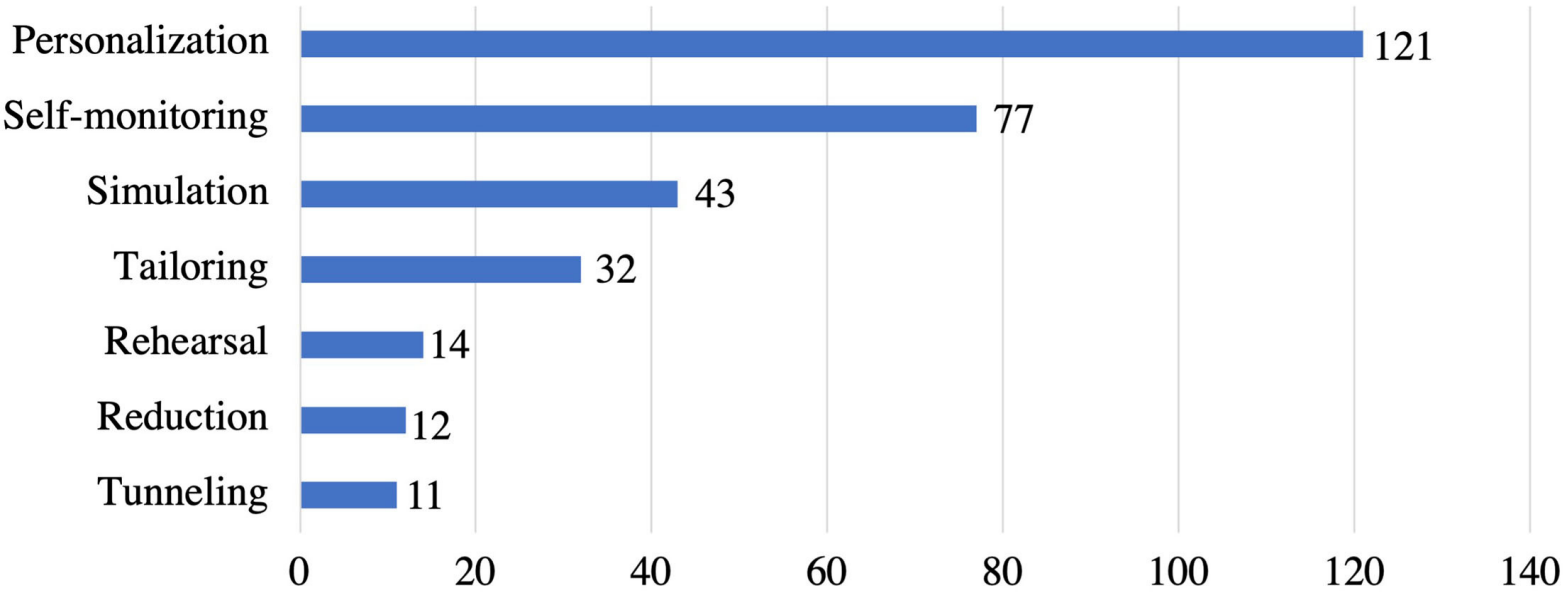
Persuasive strategies under primary task support category and the corresponding number of apps that implemented individual strategies.

Figure 4 illustrates the frequency of the primary task support strategies in the reviewed apps. Among the seven (7) strategies in primary task support, we observed that *personalization* (*n* = 121) is the most employed strategy, followed by *self-monitoring* (*n* = 77), *simulation* (*n* = 43), *tailoring* (*n* = 32), *rehearsal* (*n* = 14), and *reduction* (*n* = 12). However, *tunneling* (*n* = 11) is the least employed strategy in the primary task support category.

Each strategy was operationalized in multiple ways, as shown in Table 4. For example, *personalization* strategy was implemented via individualized pre-assessments (24 apps), personalized app content based on user's preferences (7 apps), personalized avatar (2 apps), or offering customization features (88 apps). Among the 77 apps that implemented *self-monitoring*, 17 apps utilized self-report method for recording stress and anxiety levels (using responses from pre and post-assessment questions), 33 apps provide automatic logging of stress levels using chatbots, and remaining 27 apps provided manual journaling feature to record own thoughts about your mood, stress, anxiety, etc. within the app. In addition, all the 77 apps provided some sort of visualization for users to monitor their progress using graphs/charts. Furthermore, 43 apps that implemented *simulation* strategy used the following: stress relief games (28 apps), observing stress and anxiety levels with real-time sensors (9 apps), and synchronizing activity progress to tree/flower growth (6 apps). Besides, *tailoring* was employed in a total of 32 apps, out of which 16 apps provided the option to switch languages, and the remaining 16 apps offered tailored app content based on user type and age group. On the other hand, *rehearsal* was implemented by 14 apps allowing users to practice interaction for capturing stress and anxiety levels using heart rate sensors (6 apps) and training the user about the gestures to play stress relief games (8 apps). Furthermore, 12 apps implemented *reduction* strategy by allowing users to use hashtags for quick journaling (9 apps) and a search bar option to search for specific sessions and navigate through fast (3 apps). Finally, only 11 apps implemented *tunneling* strategy by offering step-by-step weekly or daily sessions.

**Table 4:** Summary of persuasive strategies and how they are implemented in the apps.

**Strategy**	**Implementation**
**Primary task support**	
Personalization	Assessments to determine the individual's stress or anxiety level; personalized app content based on user's preferences (e.g., breathing, meditation, exam stress, work stress, panic attack, etc.); personalized avatar (e.g., customized animated face for users); Customization features (e.g., favorite list, background music, apply themes, controlling time limit for meditation sessions)
Self-monitoring	Automatic logging (e.g., using AI chatbot for recording mood status); Self-report of stress/anxiety levels using assessment questions before and after the meditation sessions; Journaling to record your own thoughts about your mood, stress, anxiety, etc. within the app; tracking progress using charts and graphs (e.g., monitor game progress; tracking session progress; improvement in stress, anxiety, etc.)
Simulation	Observing stress and anxiety levels in real-time via sensors; Simulations for stress relief in games (e.g., breaking things, fighting, hitting, etc.); synchronize activity progress with tree growth/flower growth (e.g., tree grows when user completes a milestone, flower grows petal by petal upon completing meditation sessions)
Tailoring	Tailored app content based on age groups or user types (e.g., beginners, intermediate, advanced, kids, etc.); option to switch languages
Rehearsal	Allowing user to practice the interaction for capturing stress and anxiety levels by placing finger on the heart rate sensor of the camera; Training the user about interactions and gestures to play in stress relief games
Tunneling	Step by step session milestones (e.g., progressive session tracks for meditation); Weekly milestones (e.g., 7-day track for meditation/breathing practice)
Reduction	Providing hashtags for quick logging in journal (e.g., #feelinghappy, #anxious, #stressed, etc.); search bar for searching meditation/breathing sessions in the app
**Dialogue support**	
Reminders	Personalized reminders notifying users to practice meditations, follow their goals, or log their mood; multiple notifications to maintain user engagement with the apps
Rewards	Several kinds of rewards (e.g., badges, stickers) for completing a particular number of activities, sessions, or challenges; providing advanced features (e.g., detailed statistics) when users regularly log their moods
Suggestion	Personalized recommendations (e.g., suggest different relaxation or meditation sessions, or ideas) based on pre-assessments
Praise	Praise or commendation using words after completing required levels or logging moods (e.g., “well done,” “congratulations,” “great job”)
Similarity	Adapting similar common social icons (e.g., like, comment, share, blog, or emojis)
Liking	Attractive and visually applying user interface (simple, organized)
Social role	Chat feature with specialists, listeners, or other users
**Social support**	
Social learning	Community feature (e.g., community blog for the people to share posts about the progress, success stories, etc.)
Social facilitation	Number of persons enrolled in the meditation/breathing sessions; the number of likes on the blog post
Normative influence	Chat groups for people with same purpose (e.g., anxiety relief, stress management, depression support, etc.)
Competition	Group of users competing in a game
Recognition	Leaderboard
**System credibility support**	
Trustworthiness	Providing Privacy policy/Terms & Conditions to inform users how their data is going to be used, how they can protect their data and the conditions that users should follow
Real-world feel	Listing contact and support information (e.g., email, address, phone)
Expertise	Information about the experts that created the app contents or sessions (e.g., name, photo); providing science-based contents based on expert information
Authority	Presenting developer's information, team members involved, organizations behind apps
Verifiability	Providing links about experts and content references
Surface credibility	Well-organized app content (e.g., various sessions organized to their purpose); limited ads in their free plans
Third-party endorsements	Providing information about awards, achievements, and certifications received by the app
**Cialdini's principles of persuasion**	
Reciprocity	Free trial for a brief time period (e.g., 3 days, 1 week, or 14 days)
Consistency/commitment	Customized goals for users to select or set a new goal for meditation and practicing different coping strategies (e.g., call a friend or watching a funny movie)
Scarcity	Discount coupon on annual membership for limited time (e.g., 50% discount if users subscribe in 1 week)

#### Dialogue Support Strategies and Their Implementation

**Figure 5:**
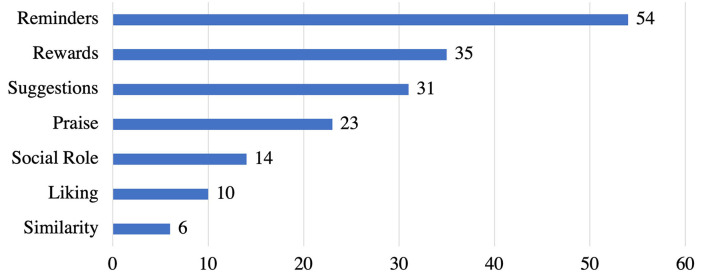
Persuasive strategies under dialogue support category and the corresponding number of apps that implemented individual strategies.

Figure 5 shows the dialogue support strategies employed in the reviewed apps. We found that *reminders* strategy (*n* = 54) is the most commonly employed strategy, followed by *rewards* (*n* = 35), *suggestion* (*n* = 31), and *praise* (*n* = 23). However, *social role* (*n* = 14), *liking* (*n* = 10), and *similarity* (*n* = 6) are the least employed dialogue support strategies in stress management apps.

Each strategy has one or more different implementations, as presented in Table 4. Out of the 54 apps that implemented the *reminders* strategy, 46 apps provide personalized reminders. Users can set times, days, and how often they want to be notified to practice meditations, follow their goals, or log their mood. Only 8 apps did not make the reminder/notification feature customizable. Furthermore, 35 apps implemented the *rewards* strategy as badges, stickers or unlock advanced features. Besides, *suggestion* was implemented as personalized recommendations in 31 apps based on the result of in-app assessments (such as questions for assessing user moods and needs). Also, 23 apps implemented *praise* as words of commendation for completing required levels or logging moods. On the other hand, *social role*, was implemented as a chat feature in 14 apps. Also, 10 apps implemented the *liking* strategy by presenting an attractive or visually appealing user interface to users. Finally, only 6 apps implemented *similarity* strategy by providing features or icons (such as emojis) that depict users' current mood.

#### Social Support Strategies and Their Implementation

**Figure 6:**
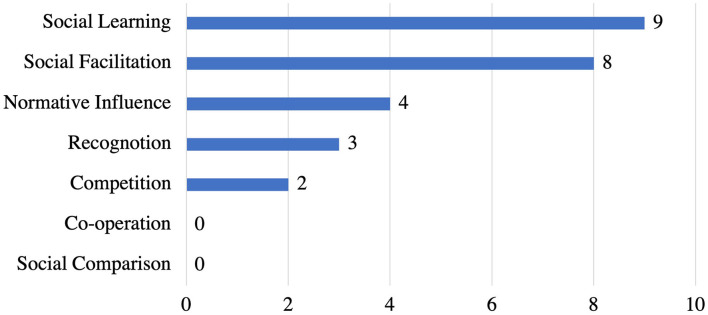
Persuasive strategies under social support category and the corresponding number of apps that implemented individual strategies.

Figure 6 illustrates the frequency of employing the social support strategies in the reviewed apps. Among the seven strategies in social support category, we uncovered that *social learning* (*n* = 9) is the most employed strategy, followed by *social facilitation* (*n* = 8), and *normative influence* (*n* = 4). Yet, *recognition* (*n* = 3) and *competition* (*n* = 2) are the least implemented strategies. While *co-operation* and *social comparison* were not implemented in any of the stress management apps reviewed.

Table 4 shows the implementations for social support strategies in the reviewed apps. *social learning* strategy was employed by 9 apps as providing users with the community blog feature for sharing their success stories so that other people can learn from them. A total of 8 apps implemented the *social facilitation* strategy by highlighting the number of enrolled users in a meditation session or the number of likes on the blog post. *Normative influence* was implemented in 4 apps as chat groups. On the other hand, *recognition* and *competition* were implemented in 3 apps (as leaderboard ranking) and 2 apps (as group of users competing in a game), respectively.

#### System Credibility Support Strategies and Their Implementation

**Figure 7:**
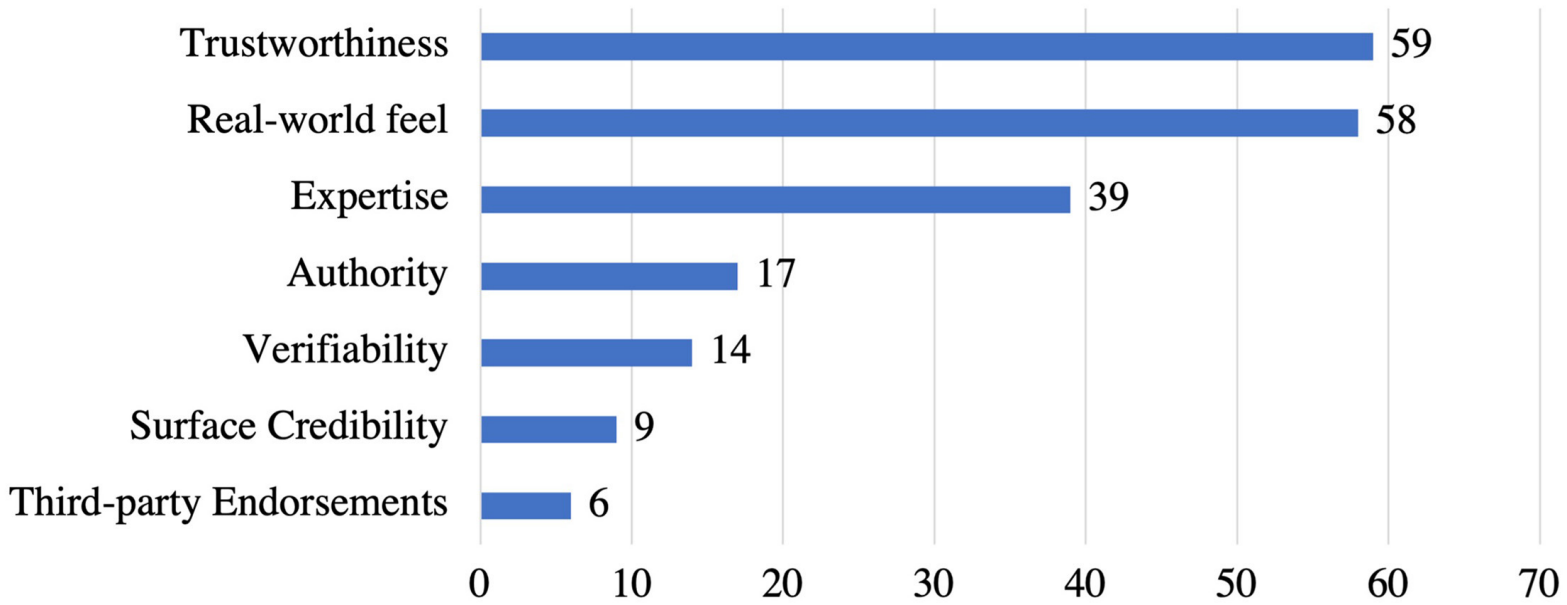
Persuasive strategies under system credibility category and the corresponding number of apps that implemented individual strategies.

Figure 7 presents the system credibility support strategies implemented in the reviewed apps. *Trustworthiness* (*n* = 59) is the most employed strategy, followed by *real-world feel* (*n* = 58), *expertise* (*n* = 39), a*uthority* (*n* = 17), and *verifiability* (*n* = 14). Yet, *surface credibility* (*n* = 9) and *third-party endorsements* (*n* = 6) are the least employed strategies in stress management apps.

Most system credibility support strategies were identified to have a single implementation, as showed in Table 4. For *trustworthiness* strategy, 59 apps provide both privacy policy and terms/conditions to explain to users how their data is going to be utilized, information about the secure data transmission to third party entities and maintaining the confidentiality of the users as a way to gain users' trust. Besides*, real-world feel* (*n* = 58) strategy was implemented by including different contact information such as e-mails, phone numbers, addresses that allow users to reach people behind the apps for providing feedback or answering their concerns. Moreover, 26 out of 39 apps implemented the *expertise* strategy by showing names, brief information, and/or photos of expertise behind the app contents or meditation sessions while 13 apps present science-based contents based on expert information. Also, 17 apps employed *authority* strategy by listing names of developers, team members involved, and the organizations such as universities behind the apps. Furthermore, 14 apps implemented *verifiability* strategy as links that show more details about experts in the app and references to verify apps' content (reference list). However, 9 apps implemented *surface credibility* strategy by presenting well-organized content according to the purpose of the apps and limited ads in their free plans. Lastly, only 6 apps implemented *third-party endorsements* strategy by showing awards, achievements, or certifications received by the app from external people or agencies.

#### CPP Persuasive Strategies and Their Implementations

**Figure 8:**
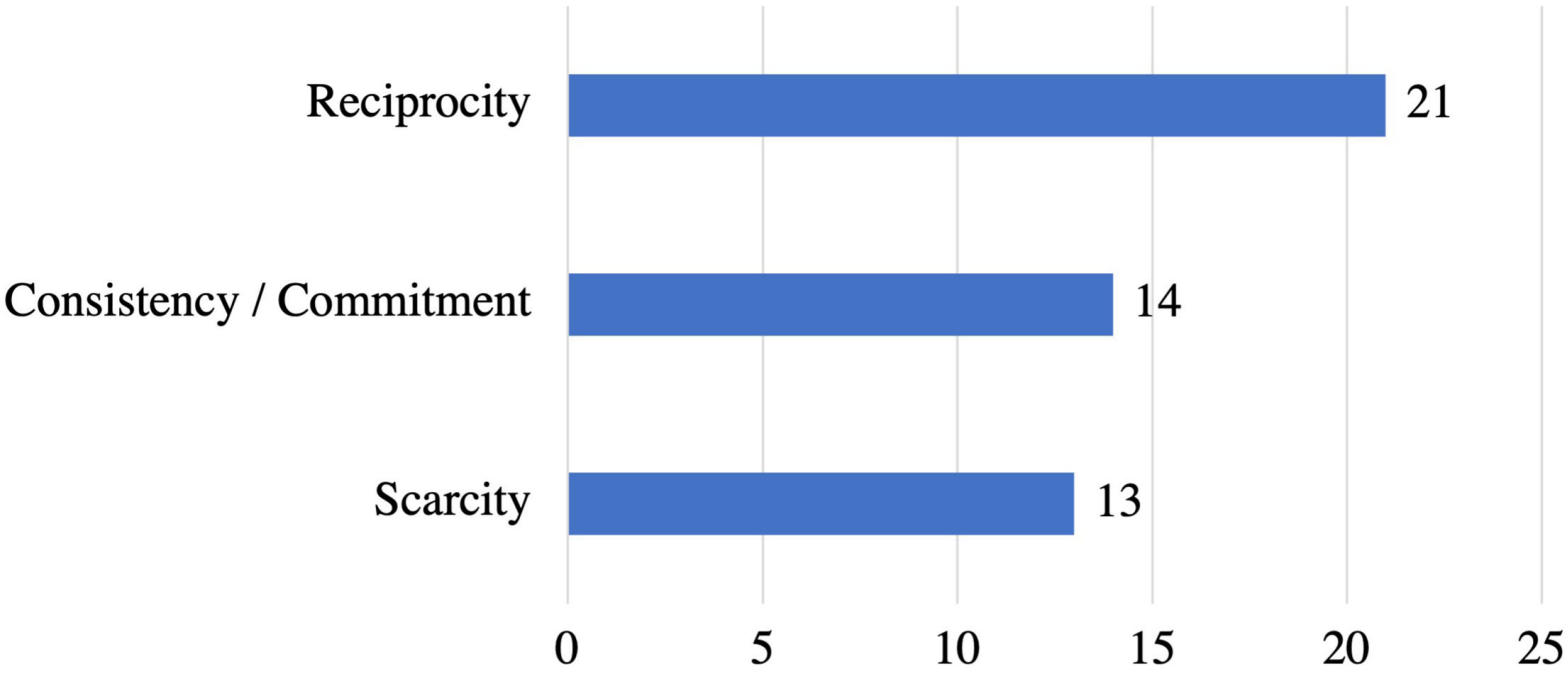
Persuasive strategies under CPP and the corresponding number of apps that implemented individual strategies.

Figure 8 shows CPP strategies employed in the reviewed apps. *Reciprocity* (*n* = 21) is the most commonly employed strategy, followed by consistency/commitment (*n* = 14). On the other hand, *scarcity* (*n* = 13) emerged as the least implemented strategy in stress apps.

Table 4 presents ways of implementation for each strategy. *Reciprocity* strategy was implemented in 21 apps by offering a free trial for a brief period such as 7 days free trial to try all advanced features in the app. Moreover, 14 apps employed *consistency/commitment* strategy by allowing users to customize their goals based on their preferences, such as setting a goal to practice specific meditation or relaxation sessions for a week. Also, users can select different goals to help them cope with their stress, such as calling a friend, watching a funny movie, or writing a letter for a family member, etc. Finally, *scarcity* is the least employed strategy, implemented in 12 apps as discount coupons on annual membership for a limited time.

### Summary of the Study Findings

**Figure 9:**
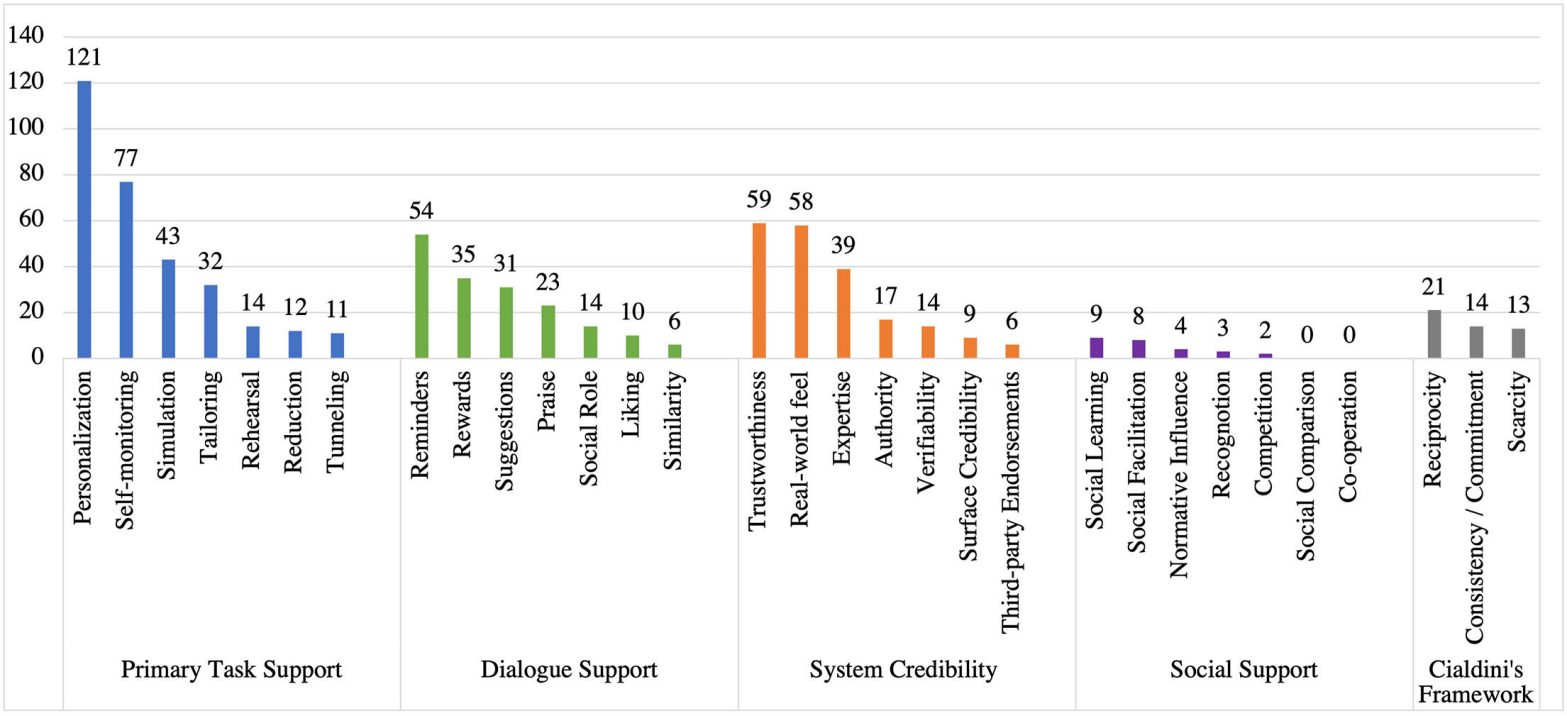
Summary of persuasive strategies employed in stress management apps.

Figure 9 shows the comparison between different categories of PSD and CPP frameworks regarding the number of persuasive strategies employed in the reviewed apps. Our findings showed that primary task support strategies of the PSD model are the most commonly employed in the reviewed apps, followed by system credibility support, dialogue support strategies, and the CPP strategies. However, we found that social support strategies are least employed overall. This answered our first research question (RQ1).

### Comparing Strategies Employed in Stress Management Apps With Other Mental Health Domains

**Figure 10:**
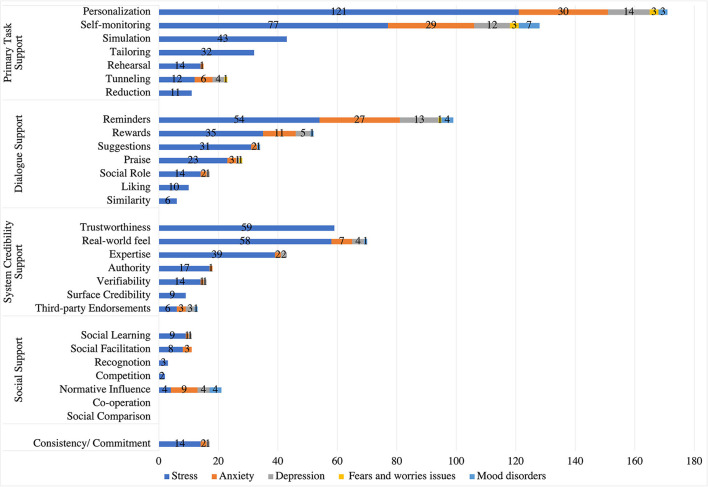
Comparing persuasive strategies in stress management apps with those in other mental health domains.

In this section, we compare our findings of persuasive strategies in stress management apps to an existing systematic review of apps in other mental health domains including anxiety, depression, fears and worries, and mood disorder (55). As shown in Figure 10, *personalization* is the most commonly implemented strategy followed by *self-monitoring* and *reminders* in anxiety apps. Similarly, *personalization* is the most frequently employed strategy, followed by *reminders* and *self-monitoring* in depression apps. *Personalization* and *self-monitoring strategies* are equally implemented in apps targeting fear and worry, followed by *reminders, tunneling*, and *praise* strategies. However, in mood disorders apps, *self-monitoring* is the most employed strategy, followed by *reminders* and *personalization*. Furthermore, *rehearsal, authority, verifiability*, and *social learning* are the least employed strategies in anxiety apps. Similarly, the least implemented strategies in depression apps are *social role, praise, verifiability*, and *social learning*. However, the least employed strategies in mood disorder are *reminders, rewards, real-world feel*, and *third-party endorsements*.

Interestingly, our findings shows that *tailoring, simulation, reduction, similarity, trustworthiness*, and *surface credibility* were not employed in any other mental health apps. This distinguishes stress-related apps from other mental health apps with respect to the strategies employed in their designs. Our finding confirms that *personalization* and *self-monitoring* are the most frequently implemented persuasive strategies in stress management and other mental health apps. On the other hand, our results show that *simulation, trustworthiness, real-world feel*, and *expertise* strategies are commonly employed in stress management apps, compared to other mental health apps. This answered our third research question (RQ3).

## Discussion

The goal of this review was to determine which persuasive strategies were employed in apps for managing stress. The following sub-sections discuss our findings based on PSD and CPP frameworks.

### Primary Support Strategies

Majority of the apps that were coded (94%) employed the *personalization* strategy to deliver content and interventions personalized to individual users based on their profiles (such as demographics, medical history, etc.) and activities within the app (i.e., behavioral patterns). Evidence already affirms the global shift from standard healthcare to personalized healthcare to enhance patient experience and significantly improve their health and wellbeing (73–75). Hence, it is unsurprising that mobile health (mHealth) apps that offer personalized content or services were found to be effective by target users (76–79), including apps for managing stress (80).

*Self-monitoring* is the next commonly used persuasive strategy after *personalization*. Our results show that 59% of the analyzed apps employed *self-monitoring* to deliver features that enable users to record their activities manually or automatically and track progress toward set goals over time. This strategy is crucial for apps that support self-management of health-related issues by enhancing users' insights into the state of their health and motivating them to take charge and seek medical attention where necessary. For example, data captured through self-monitoring can be used to track the risk of mild or chronic health conditions [including mental health issues, such as mood disorder (81), depression (82), stress (44), and others (83)] in real-time, and then trigger interventions to mitigate the risk. Interestingly, research has also found *personalization* and *self-monitoring* as dominant strategies in other mental health-related apps (55) since user activity data (or behavioral data) can be used to offer personalized services or interventions that will improve users' health and wellbeing.

Moreover, the *simulation* strategy which allows users to observe the cause and effect of their behavior was found in 34% of the analyzed apps. For example, some apps use tree and flower metaphors such as petal growth in the flower or more leaves on the tree to show the influence of completing meditation sessions on health. Research has shown that simulation can yield positive outcomes in patients experiencing stress (43, 84).

Furthermore, *tailoring* strategy offers similar content to users who share common interests and rehearsal strategy that allows users to rehearse a target behavior found in 21% of the coded apps. However, *tunneling* and *reduction* persuasive strategies were found in only 9 and 6% of the stress management apps, which is surprising since mental health patients are usually advised to avoid stressful situations and complex activities that can increase their stress levels. In addition, users can get frustrated easily if in-app support or guidance is missing in mental apps (85, 86).

### Dialogue Support Strategies

In the dialogue support category, *reminders* strategy is the most commonly employed strategy, followed by *rewards* and *suggestion*. *Reminders* strategy is the top strategy possibly because it keeps users focused on their intended tasks. For instance, users are usually reminded or notified at specific intervals to meditate, check their heart rate, or log their activities. The *suggestion* strategy offers personalized tips or recommendations as push notifications (or pop-up messages) that draw users closer to their goals. Majority of the apps that implemented the *suggestion* made it customizable such that users can enable/disable or change the timing/frequency of the notifications. This improves the users' feeling of agency and sense of control (87, 88), which can increase their motivation.

*Rewards* strategy is another motivating factor since users tend to intensify their actions if incentives are attached (89). However, offering tangible rewards to people who are already motivated could confuse them about the real motives (90) and benefits of adopting a healthy behavior (91). *Social role* is the least employed strategy based on our findings and this may reflect users' concern about privacy and trust if they share sensitive information about their mental health issues (such as stress) with conversational/virtual agents or human specialists via apps (92–94). This also mirrors research evidence that patients are generally less willing to share or engage in most personal communication while using mental health apps due to trust and privacy concerns, among other issues (95).

### System Credibility Support Strategies

Based on our findings, system credibility persuasive strategies are the second most employed strategies in stress management apps. Our results showed that *trustworthiness* strategy (39%) is the most implemented strategy followed by *real-world feel* (38% of the coded apps) and *expertise* (26% of the coded apps) in the system credibility support category of the PSD framework. From our results, we also found that *trustworthiness* was operationalized using privacy statements and informing the user about the terms and conditions for handling the collected data, which is in line with the previous research (96, 97) which shows that trustworthiness is an essential factor for increasing user engagement and safety of the personal data in the mHealth apps. In addition, (86) affirms that users using mental health apps are more concerned about their data privacy, information sharing, and data handling transparency. This explains why *trustworthiness* is the most implemented system credibility strategy in stress management apps.

According to (86), users prefer to use apps that are aesthetically pleasing (*surface credibility*) with interventions that are clinically validated (*expertise* and *third-party endorsements*) for their mental health needs. Prior research also indicates that using strategies from the system credibility category is essential for improving the overall credibility of the mHealth apps (70). In addition, apps implementing *trustworthiness, expertise*, and *authority* strategies have greater chances of persuading users to complete their target behavior (98). However, *surface credibility* and *third-party endorsements* are the least implemented strategies in the system credibility category, based on our findings. This is a call to action for app designers aiming to motivate behavior change and increase engagement in the area of stress management.

### Social Support Strategies

Although socially-driven persuasive strategies are the most frequently employed strategies in health technologies and other online support systems (64, 91, 99), we found that social support strategies are the least implemented persuasive strategies in stress management apps, as well as apps in other mental health domains (55). Most people are silent about their mental health issues possibly because they are afraid of being stigmatized if they openly discuss their health challenges (100). Consequently, they tend to withdraw from social interactions (100). Moreover, previous research has stated that it could be inappropriate to incorporate social comparison and competition in a mental health apps, particularly for stressed users since competition could induce unhealthy stress levels and discourage some people. Additionally, a positive correlation has been found between high levels of perceived competition and other mental health issues such as depression and anxiety (101). Therefore, applying *social comparison* and *competition* strategies in stress management apps could negatively impact users' stress levels.

Furthermore, literature shows that there is a relationship between cooperation and stress. According to Yuebing (102), individuals under stress worry about being exploited. Hence, they are less likely to cooperate, with tendencies to have negative expectations about their partners' behavior (102). As a result, stress may obstruct *cooperation*. However, incorporating *social learning, social facilitation*, and *normative influence* strategies in stress management apps could help stressed individuals to decrease the feeling of being alone, isolated, or stigmatized. Evidence shows that forums that provide peer support may foster social inclusion in unexpected and new ways and should be a core element of recovery-oriented mental health policy (103). In addition, peer support is a primary part of helping many people experiencing mental ill-health in their recovery journeys (104).

### CPP Strategies

Based on our findings, *reciprocity* is the most implemented strategy (21 apps), followed by *commitment* (14 apps) and *scarcity* (13 apps). This aligns with the research evidence which found reciprocity as the most implemented strategy in emotional and mental health apps (57). According to (105), if people receive something valuable in advance (such as gifts) before completing a task, they are encouraged to perform the target behavior since they feel indebted. *Commitment* is the second most commonly implemented strategy in emotional and mental health apps based on our findings and in alignment with (57). Employing *commitment* in stress management apps would motivate users to commit to their daily, weekly, or monthly goals and work consistently toward achieving them.

### Comparative Analysis

Table 5 shows the most and least dominant persuasive strategies for app category. Every individual faces a different kind of stress and the level of stress also varies from person to person. Therefore, it is not surprising that personalization is the most dominant in both “Stress Intervention Only” and “Stress Intervention and Monitoring” categories. *Self-monitoring* is essential for tracking users' stress levels and helps them visualize their progress over time. Some of the apps do not track stress levels directly, but they do track user activity, which is indirectly related to stress levels (e.g., number of meditation sessions completed by the user). This explains why the *self-monitoring* strategy is most dominant in the “Stress Monitoring Only” category.

**Table 5:** Comparative analysis of persuasive strategies across app categories (Strategies are presented in descending order of prevalence or dominance).

**Categories**	**Most dominant**	**Least dominant**
Stress Intervention Only	Personalization, Self-monitoring, Simulation, Reminders and Trustworthiness	Third-party Endorsements, Recognition, Competition, Social Comparison, and Co-operation
Stress Monitoring Only	Self-monitoring, Personalization, Trustworthiness, Expertise, and Real-world feel	Competition, Recognition, Social Comparison, Co-operation, and Consistency/commitment
Stress Monitoring and Intervention	Personalization, Self-monitoring, Real-world feel, Trustworthiness, and Reminders	Normative Influence, Competition, Recognition, Social Comparison, and Co-operation

*Trustworthiness* is another important strategy for stress management apps. Users tend to use mHealth apps that clearly specifies how their data will be stored and protected in the app. On the other hand, social support strategies (e.g., *social comparison, recognition, competition, co-operation*, etc.) are the least dominant, as the users experiencing stress are hesitant and less likely to compete with others in order to improve their stress, and most would like to keep it private to avoid stigmatization.

Furthermore, our findings revealed six (6) strategies (i.e., *tailoring, simulation, reduction, similarity, trustworthiness*, and *surface credibility*) implemented in stress management apps but not in other mental health apps. This could be due to several reasons. First, stress management apps usually *tailor* educational and interactive modules to users based on established various intervention techniques, including mindfulness, meditation, or breathing exercises (106–108). Intervention modules for stress reduction could also be tailored based on user groups such as general military members or combat-injured patients (109). Second, decreasing the complexity and steps users need to perform the target behavior (*reduction* strategy) could reduce negative emotions that contribute to high-stress levels. Third, *surface credibility* is essential for stress management apps since organized, efficient, and ads-free content could contribute to stress reduction (110). Fourth, privacy and trust concerns surrounding mental health apps, including stress (111), tend to be a major factor influencing designers' implementation of *trustworthiness* strategy in stress management apps. Finally, *simulation* strategy is widely used as a technique for stress relief such that users can see the cause and effect of their behavior. For instance, recent research, informed by Laughter therapy, implemented flower blooms at a certain point based on users' laugh energy (112). Another app encourages users to practice relaxation training that would help reduce their stress when users' stress level is balanced; a tree grows up strong and healthy when users' stress level is balanced or normal (113).

## Design Recommendations

In this section, we offer a set of design guidelines based on our review findings.

### Provide Personalized Stress Interventions

Everyone experiences varying stress levels at different times; hence, coping intervention can only produce the desired outcome if it fits each target user's current stress level. Hence, designers of stress management apps should leverage sensors data, in addition to self-reports where available, to detect stress levels in real-time using machine learning (ML) models and then offer personalized interventions that relieve the stress detected. Stress interventions can be personalized based on users' preferences through a pre-survey that introduces a list of recommended interventions, in which users can choose the interventions that work in reducing their stress—for example, practicing meditation or getting social support. In addition, users' past preferences can also be fed into ML models to determine the most suitable intervention. As a result, users will find the apps effective for managing their stress on an ongoing basis, thereby boosting user motivation and engagement/adoption in both the short and long term.

### Reduce Task Complexity and Provide In-app Support

Most reviewed stress management apps lack in-app support and fail to simplify tasks users are expected to perform. App designers should reduce tasks into simpler steps (*reduction*) and offer contextual guidance (*tunneling*) until the tasks are successfully completed to avoid raising the stress levels of their users (114). Simplifying tasks and minimizing user efforts through providing templates for journaling which users can easily fill or change as necessary. Since feeling stressed may affect users' choices, the system should automatically arrange and present interventions based on users' needs/type (*tailoring*) and support their adoption of the target intervention.

### Provide Clear Privacy Policies to Address Data Privacy Concerns

Data privacy and transparency are important factors to ensure the trustworthiness of stress-based apps (96, 97) due to the sensitive nature of the data collected. Users of mental health apps usually have privacy and trust concerns that affect their decision to use such apps (111). To establish the users' trust, designers should provide the app's clear privacy policies and clear information about the data storage. Additionally, they should also provide steps for secure data transmission to third-party entities, how the user's data is stored and utilized, and steps taken to ensure users' anonymity. This information should be presented to the user in concise and plain language, and consent obtained prior to collecting any personal data (*trustworthiness*).

### Create Apps Using Evidence-Based or Clinically Proven Techniques for Handling Stress

To improve system credibility, designers should use evidence-based or clinically-validated stress relief techniques that are proven to be effective for a larger group of audience in the design of their apps. These techniques can be in the form of daily or weekly psychoeducation materials (e.g., stress reduction, mindfulness skills, and well-being enhancement) developed by health professionals or taken from valid sources with proper references included or approved by top health authorities (e.g., World Health Organization, Public Health Agency of Canada, United States Department of Health and Human Services, etc.). Evidence-based contents or interventions delivered through mobile apps have been shown to be effective in reducing stress and improving mental wellbeing (108).

### Deliver Stable, Functional, and Professionally Looking Interfaces in the Apps

The usability of stress management apps is crucial for continuous engagement. Research has shown that aesthetically pleasing and stable user interfaces are more likely to be used by the users (86). Therefore, designers should make their apps visually attractive and have a consistent interface in terms of color, layout, and graphics. In addition, designers should ensure that the app's interface is simple, organized, easy to use/understand, and void of ambiguous and unstable functionalities (*surface credibility)*. These practices will ensure that stress management apps are appealing, credible, and motivating to users.

### Make Premium Features for Stress Intervention Accessible

Designers should provide a free trial upon user registration to allow them to use the premium features in the app. This can be achieved by offering a free trial for an initial period (e.g., 30 days) or linking the premium features to achievement of behavior change milestones (*Scarcity*). As a result, users will get the required experience to practice different stress management techniques and encourage them to continue using the app. Moreover, apps designed for stressed individuals should reduce distractions induced through in-app advertisements or ads (110).

### Allow Users to Set and Track Their Goals

Designers should provide users with an option to select their daily, weekly, or monthly goals for performing their target behavior, e.g., practicing breathing exercises or journaling their feeling/mood. In addition, designers should provide users with an activity dashboard to view progress and visualize how far they are from their defined goal. Moreover, the app should observe and monitor users' stress levels and recommend a set of specific goals to follow based on users' behavior or preferences. Research has shown that goal-setting, self-monitoring and feedback on users' performance are essential behavior change techniques (115).

### Incorporate an Online Community

Designers should include community, forum, or blog features to promote social or peer support as it is essential to overcoming stress (104, 116). Incorporating an online community will enable stressed individuals to get emotional support through empathy and other positive feelings from peers during stressful periods. Besides, users can get help or support from stress reduction techniques, advice, and suggestions shared on such in-app social settings. Therefore, accessing such social support will help users disconnect from stress by learning from other users' experiences and success stories (*social learning*).

## Limitations

This study only targeted apps developed in English languages. Also, the 150 apps analyzed were randomly selected from the pool of stress management apps extracted from both Google Play and App Store. As a result, selected apps were not uniformly distributed into the various app categories (i.e., *stress monitoring only, stress intervention only*, and *stress monitoring and intervention*). Therefore, our results may not generalize to all stress management apps but still comprehensive considering the relatively large number of apps analyzed.

## Conclusion and Future Work

In this paper, we conducted a comprehensive and comparative review of 150 stress management apps to deconstruct the persuasive strategies employed in each of them and how they were implemented. Specifically, we categorized the apps based on their purpose into three main categories: “stress intervention only,” “stress monitoring only,” and “stress intervention and monitoring.” Next, we utilized the PSD and CPP frameworks to deconstruct the persuasive or behavior change strategies utilized in each app. Our results revealed that primary task support strategies (especially *personalization* and *self-monitoring*) of the PSD model are the most commonly employed, followed by system credibility support (such as *trustworthiness* and *real-world feel*), dialogue support strategies (e.g., *reminders* and *rewards*), and the CPP strategies (e.g., *reciprocity* and *commitment/consistency*). Social support strategies are the least employed overall. We also compared stress management apps with those targeting other mental health apps to uncover further insights. Our findings revealed six (6) persuasive strategies that were solely implemented in stress management apps: *tailoring, simulation, reduction, similarity, trustworthiness*, and *surface credibility*. Finally, we offered a set of design recommendations that would inform effective, usable, engaging, and motivating apps for managing stress.

As part of future work, we will explore the strategies employed in stress management apps using additional frameworks such as the Theory of Reasoned Action/Planned Behavior and Health Belief Model. We will also examine the effectiveness of the apps based on user reviews using natural language processing and machine learning techniques.

## Data Availability Statement

The original contributions presented in the study are included in the article/Supplementary Material, further inquiries can be directed to the corresponding author/s.

## Author Contributions

MA designed the study, coded apps, analyzed the data and wrote parts of the results, discussion, and design recommendation sections. DM reviewed and coded the app, generated figures, wrote the methodology, and parts in the results section. OO validated the coding sheet of the apps, performed the statistical analysis, contributed to writing the introduction, and related work sections. NB reviewed and edited the manuscripts and contributed to writing the design recommendation section. RO guided the team through different stages of the research, reviewed and edited the manuscripts, and contributed to the writing of the discussion section. All authors contributed to manuscript revision, read, and approved the submitted version.

## Conflict of Interest

The authors declare that the research was conducted in the absence of any commercial or financial relationships that could be construed as a potential conflict of interest.

## Publisher's Note

All claims expressed in this article are solely those of the authors and do not necessarily represent those of their affiliated organizations, or those of the publisher, the editors and the reviewers. Any product that may be evaluated in this article, or claim that may be made by its manufacturer, is not guaranteed or endorsed by the publisher.
