# Supplementary material for: A Systematic and Comparative Review of Behavior Change Strategies in Stress Management Apps: Opportunities for Improvement
Source: Front Public Health. 2022 Feb 24;10:777567. doi: 10.3389/fpubh.2022.777567 (PMC8907579; doi:10.3389/fpubh.2022.777567)
Supplement: Supplementary file 1 [file Table_1.docx]

**APPENDIX A**

Following table presents the coding sheet of stress management apps.

| **App Name** | **Rating** | **Platform** | **Price** | **Language** | **Category** |
| --- | --- | --- | --- | --- | --- |
| weCare Stress Program | 3 | iPhone | Free | English | Stress Intervention Only |
| 1 Giant Mind | 4.5 | both | Free | English | Stress Monitoring and Intervention |
| 7 Cups: Anxiety & Stress Chat | 3.4 | both | Free* | English, Arabic + 20 more | Stress Intervention Only |
| 9Zest- Fix health pain stress | 3 | iPhone | Free | English | Stress Intervention Only |
| Adderss Stress | 4.4 | both | Free* | English | Stress Intervention Only |
| Adult Coloring Book For Adults Free – Mandala Pages, Stress Relief, And Color Therapy | 2.8 | iPhone | Free | English | Stress Intervention Only |
| Adult Coloring Book for Stress Releaved | No rating | iPhone | Free | English | Stress Intervention Only |
| Adult Coloring Butterfly Book For Stress Relieved | 5 | iPhone | Free | English | Stress Intervention Only |
| Adult Coloring Mandala Book For Stress Relieved | No rating | iPhone | Free | English | Stress Intervention Only |
| Adult Coloring princess girl stress relieved | 4.6 | iPhone | Free* | English + 4 more | Stress Intervention Only |
| Adult Coloring Serene Rose for stress relieved | 3 | iPhone | Free | English | Stress Intervention Only |
| Adult colouring book therapy for anti-stress | 4.5 | iPhone | Free | English | Stress Intervention Only |
| Adult Mandala colouring book therapy stress relief | 4 | iPhone | Free* | English + 2 more | Stress Intervention Only |
| Anti stress ball: DIY slime | 4.4 | iPhone | Free* | English, Russian | Stress Intervention Only |
| anti stress color therapy – adult coloring book | 2.4 | iPhone | Free* | English | Stress Intervention Only |
| Anti stress game Vacos | 3 | iPhone | Free | English | Stress Intervention Only |
| Anti stress meditation | 4.18 | android | Free | English | Stress Monitoring Only |
| Antistress – Relaxation Toys | 4.3 | both | Free* | English | Stress Intervention Only |
| AntiStress Adult Coloring Book | 5 | iPhone | Free | English | Stress Intervention Only |
| Antistress Relax: Cake on Face | No rating | both | Free | English | Stress Intervention Only |
| AntiStress Yoga SSA | 5 | iPhone | Free | English and Russian | Stress Intervention Only |
| AntiStress, Relaxing, Anxiety & Stress Relief Game | 4.1 | both | Free* | English | Stress Intervention Only |
| Anxiety Eliminator | 3 | iPhone | Free | English | Stress Intervention Only |
| Anxiety Eliminator ‚Äì Stop Stress, Stop Anxiety | No rating | both | Free* | English | Stress Intervention Only |
| Anxiety Relief Hypnosis – Stress, Panic Attacks | 4.3 | android | Free* | English | Stress Intervention Only |
| Anxiety relief pro: Stress, Panic attack help | 4.2 | android | Free* | English | Stress Intervention Only |
| Anxiety Tracker – Stress and Anxiety Log | 4.2 | android | Free* | English | Stress Monitoring Only |
| Art Break: Antistress Drawing | 4.7 | iPhone | Free* | English | Stress Intervention Only |
| Aura: Meditation & Mindfulness | 4.5 | both | Free* | English | Stress Intervention Only |
| Aurum – Stress, Anxiety, Self help, Therapy | 3 | iPhone | Free* | English | Stress Intervention Only |
| Balance Art Class: Stress Relieving Coloring Book for Adults FREE | 3.4 | iPhone | Free* | English | Stress Intervention Only |
| Beat the Boss 2 | 4.6 | iPhone | Free* | English, Danish + 15 more | Stress Intervention Only |
| Branches Stress Relief | 3 | iPhone | Free | English | Stress Intervention Only |
| Break Stuff To Relieve Stress | 3.7 | iPhone | Free* | English | Stress Intervention Only |
| Breath ball | 4.7 | Both | Free* | English | Stress Intervention Only |
| Breathly – Just Breathe | 3 | iPhone | Free | English | Stress Intervention Only |
| Brightmind: Meditation | 4.8 | Both | Free* | English | Stress Intervention Only |
| Bubble Game – Stress Relief | 3 | both | Free* | English, French and 5 more | Stress Intervention Only |
| Bubbles Antistress | 3 | iPhone | Free | English and Russian | Stress Intervention Only |
| Calm in the Storm | 4 | iPhone | Free | English | Stress Intervention Only |
| Calm: Meditate, Sleep and Relax | 4.7 | both | Free* | English, French + 4 more | Stress Intervention Only |
| Cardiac-Stress-Test | 3 | iPhone | Free | English | Stress Intervention Only |
| Colormy: Free Fun Stress Relief Color Therapy & Coloring Book for Adults | No rating | iPhone | Free* | English | Stress Intervention Only |
| Colors: Anti Stress, Relax and Sleep | 4.7 | iPhone | Free* | English + 12 more | Stress Intervention Only |
| Colors: Anti-stress,Relax,Sleep | 4.6 | android | Free* | English | Stress Intervention Only |
| Colorway – Coloring Mandala book for stress relief | No rating | iPhone | Free* | English | Stress Intervention Only |
| Colours: Anti stress, Relax, Sleep | 4.5 | android | Free | English | Stress Intervention Only |
| Community Stress First Aid | No rating | iPhone | Free | English | Stress Monitoring Only |
| Controlled Breathing Assistant | 4.6 | iPhone | Free* | English, French + 5 more | Stress Intervention Only |
| CoolStress | No rating | iPhone | Free | English | Stress Intervention Only |
| Creative Cats Art Class-Stress Relieving Coloring Books for Adults FREE | 4.8 | iPhone | Free* | English | Stress Intervention Only |
| Cure Stress | 5 | iPhone | Free | English + 29 more | Stress Intervention Only |
| Cure Stress – unique technique for relief of anxiety, pain, stress, insomnia and more | 4.5 | iPhone | Free* | English, Catalan + 20 more | Stress Intervention Only |
| Day stress relief: relaxation and and antistress app | 4.4 | android | Free | English | Stress Intervention Only |
| Daylio Journal | 4.7 | iPhone | Free | English + 28 more | Stress Intervention Only |
| DayStress Relief | 4.5 | Both | Free* | English | Stress Intervention Only |
| De-Stress: Breath & Meditation | 4.6 | iPhone | Free* | English | Stress Intervention Only |
| Deep Breath – Destress for the day | No rating | iPhone | Free* | English | Stress Intervention Only |
| Ease My Stress & Anxiety | No rating | iPhone | Free* | English | Stress Intervention Only |
| Emotional Stress Release | 3 | iPhone | Free | English | Stress Intervention Only |
| Energy: Anti Stress Loops | 4.6 | iPhone | Free | English | Stress Intervention Only |
| Exam stress | 4.2 | android | Free | English | Stress Intervention Only |
| Fidget Spinner – Office Stress Relief Toys | 3.3 | iPhone | Free* | English | Stress Intervention Only |
| Fidget Spinner – Stress Relief | No rating | both | Free | English, French + 14 more | Stress Intervention Only |
| Free Relaxing Nature scenes to reduce stress and anxiety | 4.5 | iPhone | Free* | English | Stress Intervention Only |
| FunyaFunya’s StressBank | No rating | iPhone | Free | English, Japanese | Stress Monitoring Only |
| Happify | 3.7 | both | Free* | English, Chinese + 7 more | Stress Intervention Only |
| Happy Being | 4 | iPhone | Free | English | Stress Intervention Only |
| Happy Being: Meditate, De-Stress, Sleep & well-being | 4.2 | android | Free | English | Stress Intervention Only |
| Headspace: Meditation and Sleep | 3.8 | both | Free* | English, French + 3 more | Stress Intervention Only |
| HOLD – Stress Help & Self-Care | 4.4 | both | Free | English | Stress Monitoring Only |
| HSC Stress Less | 3 | iPhone | Free | English and German | Stress Intervention Only |
| Inner Hour – Self care Therapy Anxiety and Depression | 4 | iPhone | Free* | English | Stress Intervention Only |
| Insight Timer – Meditation App | 4.8 | Both | Free* | English, Danish and 8 more | Stress Intervention Only |
| Instant Heart Rate: HR Monitor | 4.7 | both | Free* | English, Arabic and 22 more | Stress Monitoring Only |
| iRelease: guided meditation to relieve stress and increase energy instantly | 5 | iPhone | Free | English | Stress Monitoring Only |
| Kardia Deep Breathing | 4.8 | both | Free* | English, French | Stress Intervention Only |
| Leaving Stress Behind | 5 | both | Free | English | Stress Intervention Only |
| Lumosity Mind – Meditation App | 4.6 | both | Free* | English | Stress Intervention Only |
| Manage your stress | 3.7 | iPhone | Free | English | Stress Intervention Only |
| me anti stress – color therapy books for adults | 2.7 | iPhone | Free* | English | Stress Intervention Only |
| Meditopia: Meditation, Sleep | 4.8 | both | Free* | English, French + 7 more | Stress Intervention Only |
| Messed! – Silly Stress Relief | No rating | iPhone | Free | English | Stress Intervention Only |
| Mind Body Stress Relief | 5 | both | Free* | English | Stress Intervention Only |
| Mindario: Reduce Stress | 3 | iPhone | Free | English | Stress Monitoring and Intervention |
| Mindfulness Coach | 4.8 | both | Free | English | Stress Monitoring and Intervention |
| MindSurf – Manage Stress | 3.5 | both | Free* | English | Stress Intervention Only |
| Mood Fit: Stress and Anxiety | 4.8 | both | Free | English | Stress Intervention Only |
| Mood Mission: Cope up with stress, moods and anxiety | 3.2 | both | Free | English | Stress Intervention Only |
| Moving Forward | 4.5 | iPhone | Free | English | Stress Monitoring Only |
| My Stress | No rating | iPhone | Free | English | Stress Monitoring Only |
| Nature Melody ‚Äî Soothing, Calming, and Relaxing Sounds to Relieve Stress and Help Sleep Better (Free) | 5 | iPhone | Free | English, French + 29 more | Stress Intervention Only |
| Paced breathing | 4.5 | android | Free | English | Stress Intervention Only |
| Prepare for Stress | No rating | iPhone | free | English | Stress Monitoring Only |
| Rage Room : Stress Reliever | 3.8 | iPhone | Free | English | Stress Intervention Only |
| Relax Aura:Stress and worry | 5 | iPhone | Free | English | Stress Intervention Only |
| Relax Lite: Stress and Anxiety Relief | 4.6 | both | Free* | English, Chinese, French | Stress Intervention Only |
| RelaxGo – Antistress games | No rating | iPhone | Free* | English | Stress Intervention Only |
| RelaXhale – Relaxing, Calming breathing exercise to reduce stress [Free] | 3 | iPhone | Free | English | Stress Intervention Only |
| Relaxing : anti stress sound | 4.2 | android | Free | English | Stress Intervention Only |
| Relaxing Visions:Stress Aid | 3 | iPhone | Free | English + 8more | Stress Intervention Only |
| Relaxing: anti stress sounds | 4.3 | android | Free | English | Stress Intervention Only |
| Relaxing: Fluid Simulation App | 4.6 | iPhone | Free | English + 18 more | Stress Intervention Only |
| Reliefy: Antistress meditation | 4.5 | iPhone | free | English | Stress Intervention Only |
| Relieve Anxiety & Stress-Free | 3 | iPhone | Free | English | Stress Intervention Only |
| Remedy8 – Don’t Stress. Press. | 4.8 | iPhone | Free* | English | Stress Intervention Only |
| Sanity and Self anxiety stress relief sleep sounds | 4.6 | iPhone | Free* | English | Stress Intervention Only |
| Sanvello:Stress & Anxiety Help | 4.6 | both | Free* | English, Arabic and 20 more | Stress Intervention Only |
| Scratchable – Antistress Game | 4.6 | iPhone | Free* | English, Arabic + 18 more | Stress Intervention Only |
| Secret Coloring Book | 4.8 | iPhone | Free | English and Chinese | Stress Intervention Only |
| Serene – ASMR Stress Sleep Aid* | 3 | iPhone | Free* | English | Stress Intervention Only |
| Serenita – Stress & Anxiety | 4.5 | both | Free* | English, Hebrew, Traditional Chinese | Stress Monitoring and Intervention |
| Shine: Self Care and Meditation | 4.5 | iPhone | Free | English | Stress Intervention Only |
| Simple Habit: 5 Min Meditation | 4.7 | both | Free* | English | Stress Monitoring and Intervention |
| Simple Stress Survey – Stress Check | 3 | iPhone | Free | English and Japanese | Stress Monitoring Only |
| Skills, Stress Tolerance Games | 3 | iPhone | Free | English | Stress Intervention Only |
| Smash Bank Stress Relief Game | 3.3 | iPhone | Free | English | Stress Intervention Only |
| Smash Dude® | 4.4 | both | Free* | English, Chinese + 10 more | Stress Intervention Only |
| Smash Friends: Stress Buster 3D | 3 | iPhone | Free | English | Stress Intervention Only |
| Smash It! AR – Stress Relief | 4.5 | iPhone | Free* | English, Mandarin Chinese + 4 more | Stress Intervention Only |
| Smash the Mall: Stress Fix | 4.41 | android | Free | English | Stress Intervention Only |
| Spiritual Me: meditation techniques for stress relief | 4.5 | iPhone | free | English, French + 5 more | Stress Intervention Only |
| Stop, Breathe & Think | 4.8 | both | Free | English | Stress Monitoring and Intervention |
| Stress & Anxiety companion | 4.2 | android | Free | English | Stress Intervention Only |
| Stress Buster: Shooting Home | 3 | both | free | English | Stress Intervention Only |
| Stress Car | 3.4 | iPhone | Free* | English | Stress Intervention Only |
| Stress Compass | 4.5 | iPhone | Free | English, German | Stress Monitoring Only |
| Stress Flush | 3 | iPhone | Free | English | Stress Intervention Only |
| Stress Guide: HRV & Meditation | 4.7 | iPhone | Free* | English, German | Stress Monitoring Only |
| Stress Less Cards | 4.2 | Android | Free* | English | Stress Intervention Only |
| Stress Relief Adult Color Book | 3.9 | Both | free | English | Stress Intervention Only |
| Stress Relief Affirmations | 4.7 | both | Free | English | Stress Intervention Only |
| Stress Therapy | 3 | iPhone | Free | English | Stress Intervention Only |
| Stress To Joy | No rating | iPhone | free | English | Stress Intervention Only |
| Stressbusters Wellness | 4.7 | Both | free | English | Stress Intervention Only |
| StressEraser Pro | No rating | iPhone | free | English, Dutch + 3 more | Stress Monitoring Only |
| StressScan – check your stress | 4.1 | both | Free | English, Japanese + 2 more | Stress Monitoring Only |
| Take a Break! – Meditations for Stress Relief | 4.5 | iPhone | Free* | English | Stress Monitoring and Intervention |
| Total Stress Melt Meditation | 4.9 | iPhone | Free* | English | Stress Intervention Only |
| TruReach – Anxiety, Stress & Depression. | 3.6 | Both | Free* | English | Stress Monitoring and Intervention |
| Anxiety, stress and depression control | 3.5 | iPhone | free | English | Stress Monitoring Only |
| Unanxiety: Stress relief | No rating | iPhone | Free* | English, French + 7 more | Stress Intervention Only |
| Way To De-Stress | No rating | iPhone | free | English | Stress Monitoring and Intervention |
| Welltory: Heart Rate Monitor | 4.5 | Both | Free* | English, Russian | Stress Monitoring Only |
| White Noise : Calm, stress reduction, relaxing. | 4 | iPhone | Free | English + 16 more | Stress Intervention Only |
| White Noise Sleep Sounds: Rain, Nature, and more | 4.8 | iPhone | Free | English + 2 more | Stress Intervention Only |
| Wim Hof Method | 4.9 | both | Free* | English, Spanish | Stress Intervention Only |
| Wysa: stress, depression & anxiety therapy chatbot | 4.7 | both | Free* | English | Stress Intervention Only |
| Xmas Painting Book For Kids + Antistress Therapy | 3 | iPhone | Free | English, French + 29 more | Stress Intervention Only |

| **APPENDIX B**  Following table presents the coding sheet for persuasive strategy in each app | | | | | | | | | | | | | | | | | | | | | | | | | | | | | | | |
| --- | --- | --- | --- | --- | --- | --- | --- | --- | --- | --- | --- | --- | --- | --- | --- | --- | --- | --- | --- | --- | --- | --- | --- | --- | --- | --- | --- | --- | --- | --- | --- |
| **Persuasive strategies** | **Personalization** | **Self-monitoring** | **Simulation** | **Tailoring** | **Rehearsal** | **Tunneling** | **Reduction** | **Reminders** | **Rewards** | **Suggestions** | **Praise** | **Social Role** | **Liking** | **Similarity** | **Trustworthiness** | **Real-world feel** | **Expertise** | **Authority** | **Verifiability** | **Surface Credibility** | **Third-party Endorsements** | **Social Learning** | **Social Facilitation** | **Normative Influence** | **Competition** | **Recognition** | **Social Comparison** | **Co-operation** | **Reciprocity** | **Goal setting** | **Scarcity** |
| **App name** |  |  |  |  |  |  |  |  |  |  |  |  |  |  |  |  |  |  |  |  |  |  |  |  |  |  |  |  |  |  |  |
| weCare Stress Program | ✓ | ✓ |  |  |  |  |  | ✓ |  |  |  |  |  |  |  |  |  |  |  |  | ✓ |  |  |  |  |  |  |  |  |  |  |
| 1 Giant Mind |  | ✓ |  |  |  | ✓ | ✓ | ✓ | ✓ |  |  |  |  |  | ✓ | ✓ |  |  |  |  |  |  |  |  |  |  |  |  |  |  |  |
| 7 Cups: Anxiety & Stress Chat | ✓ |  |  | ✓ |  |  |  | ✓ | ✓ |  | ✓ | ✓ |  |  |  |  |  |  |  |  |  | ✓ | ✓ | ✓ |  |  |  |  |  |  |  |
| 9Zest- Fix health pain stress | ✓ |  |  |  |  |  |  | ✓ |  | ✓ | ✓ |  |  |  | ✓ | ✓ |  |  |  |  |  |  |  |  |  |  |  |  | ✓ | ✓ | ✓ |
| Adderss Stress | ✓ | ✓ |  |  | ✓ |  |  |  |  |  |  |  |  |  | ✓ |  |  |  |  |  |  |  |  |  |  |  |  |  |  |  |  |
| Adult Coloring Book For Adults Free - Mandala Pages, Stress Relief, And Color Therapy | ✓ |  | ✓ |  |  |  |  |  |  |  |  |  |  |  |  |  |  |  |  |  |  |  |  |  |  |  |  |  |  |  |  |
| Adult Coloring Book for Stress Releaved | ✓ |  | ✓ |  |  |  |  |  |  |  |  |  |  |  |  |  |  |  |  |  |  |  |  |  |  |  |  |  |  |  |  |
| Adult Coloring Butterfly Book For Stress Relieved | ✓ |  | ✓ |  |  |  |  |  |  |  |  |  |  |  |  |  |  |  |  |  |  |  |  |  |  |  |  |  |  |  |  |
| Adult Coloring Mandala Book For Stress Relieved | ✓ |  | ✓ |  |  |  |  |  |  |  |  |  |  |  |  |  |  |  |  |  |  |  |  |  |  |  |  |  |  |  |  |
| Adult Coloring princess girl stress relieved | ✓ |  |  |  |  |  |  |  |  |  |  | ✓ |  |  |  |  |  |  |  |  |  |  |  |  |  |  |  |  |  |  |  |
| Adult Coloring Serene Rose for stress relieved | ✓ |  |  |  |  |  |  |  |  |  |  | ✓ |  |  |  |  |  |  |  |  |  |  |  |  |  |  |  |  |  |  |  |
| Adult colouring book therapy for anti-stress | ✓ | ✓ |  |  |  |  |  |  |  |  |  | ✓ |  |  |  | ✓ |  |  |  |  |  |  |  |  |  |  |  |  |  |  |  |
| Adult Mandala colouring book therapy stress relief | ✓ | ✓ |  |  |  |  |  |  |  |  |  | ✓ |  |  |  |  |  |  |  |  |  |  |  |  |  |  |  |  |  |  |  |
| Anti stress ball: DIY slime | ✓ |  |  |  | ✓ |  |  |  | ✓ |  |  |  |  |  |  | ✓ |  |  |  |  |  |  |  |  |  |  |  |  |  |  |  |
| anti stress color therapy - adult coloring book |  |  |  |  |  |  |  |  |  |  |  |  |  |  |  |  |  |  |  |  |  |  |  |  |  |  |  |  |  |  |  |
| Anti stress game Vacos | ✓ | ✓ |  |  |  | ✓ |  |  | ✓ |  |  |  |  |  | ✓ |  |  |  |  |  |  |  |  |  |  |  |  |  |  |  |  |
| Anti stress meditation | ✓ |  |  |  |  |  |  |  |  |  |  |  |  |  |  |  |  |  |  |  |  |  |  |  |  |  |  |  |  |  |  |
| Antistress - Relaxation Toys |  |  | ✓ |  | ✓ |  |  |  |  |  |  |  | ✓ |  |  | ✓ |  |  |  |  |  |  |  |  |  |  |  |  |  |  |  |
| AntiStress Adult Coloring Book | ✓ |  | ✓ |  |  |  |  |  | ✓ |  |  |  |  |  |  | ✓ |  |  |  |  |  |  |  |  | ✓ | ✓ |  |  | ✓ |  |  |
| Antistress Relax: Cake on Face | ✓ |  |  |  |  | ✓ |  |  |  |  | ✓ |  |  |  | ✓ |  |  |  |  |  |  |  |  |  |  |  |  |  |  |  |  |
| AntiStress Yoga SSA | ✓ |  |  | ✓ |  |  |  | ✓ |  |  |  |  |  |  | ✓ |  |  |  |  |  |  |  |  |  |  |  |  |  |  |  |  |
| AntiStress, Relaxing, Anxiety & Stress Relief Game | ✓ |  | ✓ | ✓ |  |  |  |  |  |  |  |  |  |  |  |  |  |  |  |  |  |  |  |  |  |  |  |  |  |  |  |
| Anxiety Eliminator | ✓ |  |  |  |  |  |  |  |  |  |  |  |  |  |  | ✓ | ✓ |  |  |  |  |  |  |  |  |  |  |  |  |  |  |
| Anxiety Eliminator ‚Äì Stop Stress, Stop Anxiety |  |  |  |  |  |  |  |  |  |  |  |  |  |  |  | ✓ |  |  | ✓ |  |  |  |  |  |  |  |  |  |  |  |  |
| Anxiety Relief Hypnosis - Stress, Panic Attacks | ✓ |  |  |  |  |  | ✓ |  |  |  |  |  |  |  |  | ✓ |  |  |  |  |  |  |  |  |  |  |  |  |  |  |  |
| Anxiety relief pro: Stress, Panic attack help | ✓ |  |  |  |  |  |  |  |  |  |  |  |  |  |  |  |  |  |  |  |  |  |  |  |  |  |  |  |  |  |  |
| Anxiety Tracker - Stress and Anxiety Log | ✓ | ✓ |  | ✓ |  |  |  | ✓ |  |  |  |  |  |  | ✓ |  | ✓ |  | ✓ |  |  |  |  |  |  |  |  |  |  |  |  |
| Art Break |  |  |  |  |  |  |  |  |  |  |  |  |  | ✓ | ✓ |  |  |  |  |  |  |  |  |  |  |  |  |  |  |  |  |
| Art Break: Antistress Drawing | ✓ | ✓ |  |  |  |  | ✓ |  |  |  |  |  |  |  | ✓ | ✓ |  |  |  |  |  |  |  |  |  |  |  |  |  |  |  |
| Aura: Meditation & Mindfulness | ✓ | ✓ |  | ✓ |  |  |  | ✓ | ✓ |  | ✓ |  |  |  | ✓ | ✓ |  |  |  | ✓ |  |  | ✓ |  |  |  |  |  | ✓ |  |  |
| Aurum - Stress, Anxiety, Self help, Therapy | ✓ | ✓ |  |  |  |  |  | ✓ | ✓ | ✓ | ✓ | ✓ |  |  | ✓ | ✓ | ✓ |  |  |  |  |  |  |  |  |  |  |  |  |  |  |
| Balance Art Class: Stress Relieving Coloring Book for Adults FREE |  |  |  |  |  |  |  |  |  |  |  |  |  |  |  |  |  |  |  |  |  |  |  |  |  |  |  |  |  |  |  |
| Beat the Boss 2 | ✓ |  | ✓ |  |  |  |  |  | ✓ |  | ✓ |  |  |  |  |  |  |  |  |  |  |  |  |  |  |  |  |  |  |  |  |
| Branches Stress Relief | ✓ | ✓ | ✓ |  |  |  |  |  |  | ✓ |  |  | ✓ |  |  |  | ✓ |  | ✓ | ✓ |  |  |  |  |  |  |  |  |  | ✓ |  |
| Break Stuff To Relieve Stress |  | ✓ | ✓ |  |  |  |  |  |  |  |  |  |  |  |  |  |  |  |  |  |  |  |  |  |  |  |  |  |  |  |  |
| Breath ball | ✓ |  |  |  | ✓ |  |  |  |  |  |  |  |  |  | ✓ |  | ✓ |  |  |  |  |  |  |  |  |  |  |  |  |  |  |
| Breathly - Just Breathe | ✓ | ✓ |  |  |  |  |  | ✓ |  | ✓ |  |  |  |  | ✓ | ✓ |  |  |  |  |  |  |  |  |  |  |  |  |  |  |  |
| Brightmind: Meditation | ✓ | ✓ |  |  |  | ✓ |  |  | ✓ | ✓ | ✓ |  |  |  | ✓ |  | ✓ |  |  |  |  |  |  | ✓ |  |  |  |  | ✓ |  |  |
| Bubble Game - Stress Relief | ✓ | ✓ | ✓ |  |  |  |  |  |  |  |  |  |  |  |  |  |  |  |  |  |  |  |  |  |  |  |  |  |  |  |  |
| Bubbles Antistress | ✓ |  | ✓ |  |  |  |  |  |  |  |  |  |  |  | ✓ |  |  |  |  |  |  |  |  |  |  |  |  |  |  |  |  |
| Calm in the Storm | ✓ | ✓ |  |  |  |  |  |  |  | ✓ | ✓ |  |  |  |  |  |  | ✓ |  |  |  |  |  |  |  |  |  |  |  |  |  |
| Calm: Meditate, Sleep and Relax | ✓ |  | ✓ | ✓ |  |  |  |  | ✓ | ✓ | ✓ |  |  | ✓ | ✓ |  | ✓ |  |  |  |  |  |  |  |  |  |  |  | ✓ |  |  |
| Cardiac-Stress-Test |  |  |  |  |  |  |  |  |  |  |  |  |  |  |  |  | ✓ | ✓ | ✓ |  |  |  |  |  |  |  |  |  |  |  |  |
| Colormy: Free Fun Stress Relief Color Therapy & Coloring Book for Adults |  |  |  |  |  |  |  |  |  |  |  |  |  |  |  |  |  |  |  |  |  |  |  |  |  |  |  |  |  |  |  |
| Colors: Anti-stress,Relax,Sleep | ✓ | ✓ | ✓ |  | ✓ |  |  | ✓ |  |  |  |  |  |  |  | ✓ | ✓ | ✓ | ✓ |  |  |  |  |  |  |  |  |  |  |  |  |
| Colorway - Coloring Mandala book for stress relief |  |  |  |  |  |  |  |  |  |  |  |  |  |  |  |  |  |  |  |  |  |  |  |  |  |  |  |  |  |  |  |
| Colours: Anti stress, Relax, Sleep |  |  |  |  |  |  |  |  |  |  |  |  |  |  |  |  |  |  |  |  |  |  |  |  |  |  |  |  |  |  |  |
| Community Stress First Aid |  |  |  |  |  |  |  |  |  |  |  |  |  |  |  |  |  |  |  |  |  |  |  |  |  |  |  |  |  |  |  |
| Controlled Breathing Assistant | ✓ | ✓ |  |  |  |  |  | ✓ |  |  |  |  |  |  |  |  |  |  |  |  |  |  |  |  |  |  |  |  |  |  |  |
| CoolStress |  |  |  |  |  |  |  | ✓ |  |  |  | ✓ |  |  | ✓ |  |  | ✓ | ✓ |  |  | ✓ |  |  |  |  |  |  |  |  |  |
| Creative Cats Art Class-Stress Relieving Coloring Books for Adults FREE |  |  |  |  |  |  |  |  |  |  |  |  |  |  |  |  |  |  |  |  |  |  |  |  |  |  |  |  |  |  |  |
| Cure Stress | ✓ |  |  | ✓ |  | ✓ |  | ✓ |  |  |  |  |  |  |  | ✓ | ✓ |  |  |  |  |  |  |  |  |  |  |  |  |  |  |
| Cure Stress - unique technique for relief of anxiety, pain, stress, insomnia and more |  |  |  |  |  |  |  |  |  |  |  |  |  |  |  |  |  |  |  |  |  |  |  |  |  |  |  |  |  |  |  |
| Day stress relief: relaxation and and antistress app | ✓ | ✓ |  |  |  |  |  |  |  |  |  |  |  |  | ✓ | ✓ |  |  |  |  |  |  |  |  |  |  |  |  | ✓ |  |  |
| Daylio Journal | ✓ | ✓ |  |  |  |  | ✓ | ✓ | ✓ | ✓ |  | ✓ | ✓ |  | ✓ | ✓ |  |  |  |  |  |  |  |  |  |  |  |  | ✓ | ✓ | ✓ |
| DayStress Relief | ✓ | ✓ | ✓ | ✓ |  |  |  | ✓ | ✓ | ✓ |  |  |  |  | ✓ |  |  |  |  |  |  |  |  |  |  |  |  |  | ✓ |  | ✓ |
| Deep Breath - Destress for the day |  |  |  |  |  |  |  | ✓ |  |  | ✓ |  |  |  |  |  |  |  |  |  |  |  |  |  |  |  |  |  |  |  |  |
| De-Stress: Breath & Meditation |  | ✓ |  | ✓ |  | ✓ |  |  |  |  |  |  |  |  |  | ✓ |  |  |  |  |  |  |  |  |  |  |  |  | ✓ |  |  |
| Ease My Stress & Anxiety | ✓ | ✓ | ✓ | ✓ | ✓ |  |  | ✓ |  | ✓ | ✓ |  |  |  | ✓ | ✓ |  |  |  |  |  |  |  |  |  |  |  |  | ✓ |  |  |
| Emotional Stress Release | ✓ | ✓ |  |  |  |  |  |  |  |  |  | ✓ |  |  |  |  |  |  |  |  |  |  |  |  |  |  |  |  |  |  |  |
| Energy: Anti Stress Loops | ✓ | ✓ | ✓ |  |  |  | ✓ | ✓ | ✓ |  |  |  |  |  | ✓ |  |  |  |  |  |  |  |  |  |  | ✓ |  |  |  |  |  |
| Exam stress | ✓ |  |  |  |  |  |  |  |  |  |  |  |  |  |  |  |  | ✓ |  |  |  |  |  |  |  |  |  |  |  |  |  |
| Fidget Spinner - Office Stress Relief Toys |  | ✓ | ✓ |  |  |  |  |  |  |  |  |  |  |  |  |  |  |  |  |  |  |  |  |  |  |  |  |  |  |  |  |
| Fidget Spinner - Stress Relief | ✓ | ✓ | ✓ |  |  |  |  |  |  |  |  |  |  |  |  |  |  |  |  |  |  |  |  |  |  |  |  |  |  |  |  |
| Free Relaxing Nature scenes to reduce stress and anxiety | ✓ |  |  |  |  |  |  |  |  |  |  |  |  |  | ✓ | ✓ |  |  |  |  |  |  |  |  |  |  |  |  |  |  |  |
| FunyaFunya's StressBank | ✓ |  | ✓ |  |  |  |  |  |  |  | ✓ |  |  |  |  |  |  |  |  |  |  |  |  |  |  |  |  |  |  |  |  |
| Happify | ✓ | ✓ |  | ✓ |  |  |  | ✓ | ✓ | ✓ |  |  |  |  |  |  | ✓ |  |  | ✓ |  | ✓ |  | ✓ |  |  |  |  |  | ✓ | ✓ |
| Happy Being | ✓ | ✓ |  |  |  |  |  | ✓ |  | ✓ |  | ✓ |  |  |  |  |  |  |  |  |  |  |  |  |  |  |  |  |  |  |  |
| Happy Being: Meditate, De-Stress, Sleep & well-being | ✓ | ✓ |  |  |  |  |  | ✓ |  | ✓ |  | ✓ |  |  |  |  |  |  |  |  |  |  |  |  |  |  |  |  |  | ✓ | ✓ |
| Headspace: Meditation and Sleep | ✓ | ✓ | ✓ | ✓ |  |  |  | ✓ |  | ✓ |  | ✓ |  |  | ✓ | ✓ | ✓ |  |  |  |  |  |  |  |  |  |  |  | ✓ |  |  |
| HOLD - Stress Help & Self-Care | ✓ | ✓ |  | ✓ |  |  | ✓ | ✓ |  |  |  |  |  |  | ✓ |  |  |  |  |  |  |  |  |  |  |  |  |  |  |  |  |
| HSC Stress Less | ✓ |  |  |  |  |  |  | ✓ |  | ✓ |  |  |  |  |  | ✓ |  |  |  |  |  |  |  |  |  |  |  |  |  |  |  |
| Inner Hour - Self care Therapy Anxiety and Depression | ✓ | ✓ |  | ✓ |  |  |  | ✓ | ✓ | ✓ |  | ✓ |  |  | ✓ |  |  | ✓ |  |  |  |  |  |  |  |  |  |  |  | ✓ |  |
| Insight Timer - Meditation App | ✓ |  |  | ✓ |  |  |  | ✓ |  |  | ✓ |  |  |  | ✓ |  | ✓ |  |  |  |  | ✓ | ✓ |  |  |  |  |  | ✓ |  |  |
| Instant Heart Rate: HR Monitor | ✓ | ✓ |  |  |  | ✓ |  | ✓ |  |  |  |  |  |  |  | ✓ |  |  |  |  |  | ✓ |  |  |  |  |  |  |  |  | ✓ |
| iRelease: guided meditation to relieve stress and increase energy instantly | ✓ | ✓ | ✓ | ✓ |  |  |  |  |  |  |  |  |  |  | ✓ | ✓ | ✓ |  |  |  |  |  |  |  |  |  |  |  |  |  |  |
| Kardia Deep Breathing | ✓ |  |  | ✓ |  |  |  |  |  |  |  |  |  |  | ✓ | ✓ |  |  |  |  |  |  |  |  |  |  |  |  | ✓ |  |  |
| Leaving Stress Behind |  |  |  |  |  |  | ✓ |  |  |  |  |  |  | ✓ |  | ✓ | ✓ | ✓ |  |  | ✓ |  | ✓ |  |  |  |  |  |  |  |  |
| Lumosity Mind - Meditation App | ✓ | ✓ |  |  |  | ✓ |  | ✓ |  |  | ✓ |  | ✓ |  | ✓ | ✓ |  |  |  | ✓ |  |  |  |  |  |  |  |  |  |  |  |
| Manage your stress | ✓ | ✓ |  |  |  |  |  |  |  |  |  |  |  |  |  |  |  |  |  |  |  |  |  |  |  |  |  |  |  |  |  |
| me anti stress - color therapy books for adults | ✓ |  | ✓ |  |  |  |  |  |  |  |  |  |  |  |  |  |  |  |  |  |  |  |  |  |  |  |  |  |  |  |  |
| Meditopia: Meditation, Sleep | ✓ | ✓ |  | ✓ |  |  | ✓ | ✓ | ✓ |  | ✓ |  | ✓ |  |  | ✓ |  |  |  |  |  |  | ✓ |  | ✓ |  |  |  | ✓ |  |  |
| Messed! - Silly Stress Relief | ✓ |  |  |  |  |  |  |  |  |  |  |  |  |  |  |  |  |  |  |  |  |  |  |  |  |  |  |  |  |  |  |
| Mind Body Stress Relief | ✓ | ✓ |  |  |  |  |  |  |  |  |  |  |  |  | ✓ |  | ✓ |  |  |  |  |  |  |  |  |  |  |  |  | ✓ |  |
| Mindario: Reduce Stress | ✓ | ✓ |  | ✓ |  |  |  |  |  |  |  |  |  |  | ✓ | ✓ |  |  |  |  |  |  |  |  |  |  |  |  |  |  |  |
| Mindfulness Coach | ✓ | ✓ | ✓ |  |  |  | ✓ | ✓ | ✓ | ✓ |  |  | ✓ |  | ✓ | ✓ | ✓ | ✓ | ✓ | ✓ |  |  |  |  |  |  |  |  |  | ✓ |  |
| MindSurf - Manage Stress | ✓ | ✓ |  |  |  |  |  | ✓ |  |  |  |  |  |  |  | ✓ |  |  |  |  |  |  |  |  |  |  |  |  |  | ✓ |  |
| Mood Fit: Stress and Anxiety | ✓ | ✓ |  |  |  |  | ✓ | ✓ |  | ✓ |  |  | ✓ | ✓ | ✓ | ✓ |  |  |  | ✓ |  |  |  |  |  |  |  |  |  | ✓ |  |
| Mood Mission: Cope up with stress, moods and anxiety | ✓ | ✓ |  |  |  |  |  |  | ✓ | ✓ | ✓ |  |  |  |  |  | ✓ | ✓ | ✓ |  | ✓ |  |  |  |  |  |  |  |  | ✓ |  |
| Moving Forward | ✓ | ✓ |  |  |  |  |  |  |  |  |  |  |  |  | ✓ | ✓ | ✓ | ✓ |  |  | ✓ |  |  |  |  |  |  |  |  |  |  |
| My Stress |  | ✓ |  | ✓ |  |  |  | ✓ |  |  |  |  |  |  |  | ✓ |  | ✓ | ✓ |  |  |  |  |  |  |  |  |  |  |  |  |
| Nature Melody ‚Äî Soothing, Calming, and Relaxing Sounds to Relieve Stress and Help Sleep Better (Free) | ✓ |  |  |  |  |  |  | ✓ |  |  |  |  |  |  |  | ✓ |  |  |  |  |  |  |  |  |  |  |  |  |  |  |  |
| Paced breathing | ✓ | ✓ | ✓ | ✓ |  |  |  |  |  | ✓ |  |  |  |  | ✓ | ✓ | ✓ |  |  |  |  |  |  |  |  |  |  |  |  |  |  |
| Prepare for Stress | ✓ | ✓ |  |  |  |  |  |  |  |  |  |  |  |  |  |  |  |  |  |  |  |  |  |  |  |  |  |  |  |  |  |
| Rage Room : Stress Reliever | ✓ |  | ✓ |  |  |  |  |  | ✓ |  |  |  |  |  | ✓ | ✓ |  |  |  |  |  |  |  |  |  |  |  |  |  |  |  |
| Relax Aura:Stress and worry | ✓ |  |  |  |  |  |  |  |  |  |  |  |  |  |  | ✓ |  |  |  |  |  |  |  |  |  |  |  |  |  |  |  |
| Relax Lite: Stress and Anxiety Relief | ✓ |  |  | ✓ |  | ✓ |  |  | ✓ |  |  |  |  |  |  |  |  |  | ✓ |  |  |  |  |  |  |  |  |  |  |  |  |
| RelaxGo - Antistress games |  |  | ✓ |  |  |  |  | ✓ | ✓ | ✓ |  |  |  |  | ✓ |  |  |  |  |  |  |  |  |  |  |  |  |  | ✓ |  |  |
| RelaXhale - Relaxing, Calming breathing exercise to reduce stress [Free] | ✓ |  |  |  | ✓ |  |  | ✓ |  |  |  |  |  |  |  |  |  |  |  |  |  |  |  |  |  |  |  |  |  |  |  |
| Relaxing : anti stress sound | ✓ |  |  |  |  |  |  |  |  |  |  |  |  |  |  |  |  |  |  |  |  |  |  |  |  |  |  |  |  |  |  |
| Relaxing Visions:Stress Aid | ✓ |  |  |  |  |  |  | ✓ |  |  |  |  |  |  |  |  |  |  |  |  |  |  |  |  |  |  |  |  |  |  |  |
| Relaxing: anti stress sounds | ✓ |  |  |  |  |  |  |  |  |  |  |  |  |  |  |  |  |  |  |  |  |  |  |  |  |  |  |  |  |  |  |
| Relaxing: Fluid Simulation App | ✓ | ✓ | ✓ |  |  |  |  |  |  |  |  |  |  |  | ✓ | ✓ |  |  |  |  |  |  |  |  |  |  |  |  | ✓ |  | ✓ |
| Reliefy: Antistress meditation | ✓ |  |  |  |  |  |  | ✓ |  |  |  |  | ✓ |  | ✓ | ✓ |  |  |  | ✓ |  |  |  |  |  |  |  |  | ✓ |  | ✓ |
| Relieve Anxiety & Stress-Free | ✓ |  |  |  |  |  |  |  |  |  |  |  |  |  |  | ✓ | ✓ |  |  |  |  |  |  |  |  |  |  |  |  |  |  |
| Remedy8 - Don't Stress. Press. | ✓ | ✓ |  |  |  |  |  |  |  |  |  |  |  |  |  |  |  |  |  |  |  |  |  |  |  |  |  |  |  |  |  |
| Sanity and Self anxiety stress relief sleep sounds | ✓ | ✓ |  |  |  |  |  | ✓ | ✓ | ✓ |  |  |  |  | ✓ | ✓ | ✓ | ✓ |  |  |  | ✓ | ✓ |  |  |  |  |  |  |  |  |
| Sanvello:Stress & Anxiety Help | ✓ | ✓ |  |  |  | ✓ |  | ✓ |  | ✓ |  | ✓ |  | ✓ | ✓ | ✓ | ✓ |  |  |  |  | ✓ |  | ✓ |  |  |  |  |  | ✓ |  |
| Scratchable - Antistress Game | ✓ |  | ✓ |  |  |  |  |  | ✓ |  | ✓ |  |  |  |  |  |  |  |  |  |  |  |  |  |  |  |  |  | ✓ |  |  |
| Secret Coloring Book | ✓ | ✓ | ✓ |  |  |  |  |  |  |  |  |  |  |  |  |  |  |  |  |  |  |  |  |  |  |  |  |  |  |  |  |
| Serene - ASMR Stress Sleep Aid* | ✓ |  |  | ✓ |  |  |  | ✓ |  |  |  |  |  |  |  |  | ✓ |  |  |  |  |  |  |  |  |  |  |  |  |  |  |
| Serenita - Stress & Anxiety |  |  |  |  |  |  |  |  |  |  |  |  |  |  |  |  |  |  |  |  |  |  |  |  |  |  |  |  |  |  |  |
| Shine: Self Care and Meditation | ✓ | ✓ |  | ✓ |  |  | ✓ | ✓ |  | ✓ | ✓ |  | ✓ | ✓ | ✓ | ✓ | ✓ |  |  |  |  | ✓ | ✓ |  |  |  |  |  | ✓ |  |  |
| Simple Habit: 5 Min Meditation | ✓ | ✓ |  |  |  |  |  | ✓ | ✓ | ✓ |  |  |  |  | ✓ | ✓ |  |  |  |  | ✓ | ✓ | ✓ |  |  |  |  |  | ✓ |  |  |
| Simple Stress Survey - Stress Check | ✓ | ✓ |  |  |  |  |  |  |  |  |  |  |  |  |  |  |  |  |  |  |  |  |  |  |  |  |  |  |  |  |  |
| Skills, Stress Tolerance Games | ✓ | ✓ |  |  | ✓ |  |  |  |  |  | ✓ |  |  |  | ✓ | ✓ | ✓ |  | ✓ |  |  |  |  |  |  |  |  |  |  |  | ✓ |
| Smash Bank Stress Relief Game | ✓ |  |  |  |  |  |  |  | ✓ |  |  |  |  |  | ✓ |  |  |  |  |  |  |  |  |  |  | ✓ |  |  |  |  |  |
| Smash Dude® | ✓ |  | ✓ | ✓ | ✓ |  |  |  | ✓ |  |  |  |  |  | ✓ |  |  |  |  |  |  |  |  |  |  |  |  |  |  |  |  |
| Smash Friends: Stress Buster 3D | ✓ | ✓ | ✓ |  |  |  |  |  | ✓ |  |  |  |  |  |  |  |  |  |  |  |  |  |  |  |  |  |  |  |  |  |  |
| Smash It! AR - Stress Relief | ✓ |  | ✓ | ✓ |  |  |  |  |  |  |  |  |  |  |  |  |  |  |  |  |  |  |  |  |  |  |  |  |  |  |  |
| Smash the Mall: Stress Fix | ✓ | ✓ | ✓ |  |  |  |  |  | ✓ |  | ✓ |  |  |  |  |  |  |  |  |  |  |  |  |  |  |  |  |  |  |  |  |
| Spiritual Me: meditation techniques for stress relief | ✓ | ✓ |  | ✓ |  |  |  | ✓ |  | ✓ |  |  |  |  | ✓ | ✓ |  |  |  |  |  |  |  |  |  |  |  |  |  |  |  |
| Stop, Breathe & Think | ✓ | ✓ |  | ✓ |  |  | ✓ | ✓ | ✓ | ✓ |  |  |  |  | ✓ | ✓ | ✓ | ✓ |  | ✓ |  |  |  |  |  |  |  |  |  |  | ✓ |
| Stress & Anxiety companion | ✓ | ✓ |  |  |  |  |  | ✓ |  | ✓ |  |  |  |  |  |  | ✓ |  |  |  |  |  |  |  |  |  |  |  |  |  |  |
| Stress Buster: Shooting Home | ✓ | ✓ | ✓ |  |  |  |  |  |  |  | ✓ |  |  |  |  |  |  |  |  |  |  |  |  |  |  |  |  |  |  |  |  |
| Stress Car | ✓ | ✓ | ✓ |  |  |  |  |  | ✓ |  | ✓ |  |  |  | ✓ |  |  |  |  |  |  |  |  |  |  |  |  |  |  |  |  |
| Stress Compass |  | ✓ | ✓ |  |  |  |  |  |  |  |  |  |  |  | ✓ |  |  |  |  |  |  |  |  |  |  |  |  |  |  |  |  |
| Stress Flush | ✓ |  |  |  |  |  |  |  |  | ✓ |  |  |  |  |  |  | ✓ | ✓ |  |  |  |  |  |  |  |  |  |  |  |  |  |
| Stress Guide: HRV & Meditation |  | ✓ | ✓ |  |  |  |  |  | ✓ |  |  |  |  |  | ✓ | ✓ | ✓ | ✓ |  |  | ✓ |  |  |  |  |  |  |  |  | ✓ |  |
| Stress Less Cards | ✓ |  |  |  |  |  |  | ✓ |  |  |  |  |  |  | ✓ |  | ✓ |  |  |  |  |  |  |  |  |  |  |  |  |  |  |
| Stress Relief Adult Color Book | ✓ |  | ✓ | ✓ |  |  |  |  |  |  |  |  |  |  | ✓ |  |  |  | ✓ |  |  |  |  |  |  |  |  |  |  |  |  |
| Stress Relief Affirmations | ✓ |  |  |  | ✓ |  |  | ✓ | ✓ |  |  |  |  |  |  |  | ✓ |  |  |  |  |  |  |  |  |  |  |  |  |  | ✓ |
| Stress Therapy | ✓ | ✓ | ✓ |  |  |  |  | ✓ | ✓ | ✓ |  |  |  |  | ✓ | ✓ |  |  |  |  |  |  |  |  |  |  |  |  |  |  |  |
| Stress To Joy |  | ✓ |  |  |  |  |  |  |  |  |  |  |  |  |  |  | ✓ | ✓ |  |  |  |  |  |  |  |  |  |  |  |  |  |
| Stressbusters Wellness | ✓ | ✓ |  |  | ✓ |  |  | ✓ | ✓ |  |  |  |  |  |  | ✓ | ✓ | ✓ |  |  |  |  |  |  |  |  |  |  |  | ✓ |  |
| StressEraser Pro | ✓ | ✓ |  |  | ✓ |  |  |  |  |  |  |  |  |  |  | ✓ | ✓ |  | ✓ |  |  |  |  |  |  |  |  |  |  |  |  |
| StressScan - check your stress |  | ✓ | ✓ |  |  |  |  | ✓ |  |  |  |  |  |  |  | ✓ |  |  |  |  |  |  |  |  |  |  |  |  |  |  |  |
| Take a Break! - Meditations for Stress Relief | ✓ |  |  |  |  |  |  |  |  |  |  |  |  |  |  | ✓ |  |  |  |  |  |  |  |  |  |  |  |  |  |  |  |
| Total Stress Melt Meditation | ✓ |  |  |  |  |  |  | ✓ |  |  |  |  |  |  |  | ✓ |  |  |  |  |  |  |  |  |  |  |  |  |  |  |  |
| TruReach - Anxiety, Stress & Depression. |  |  |  |  |  |  |  |  |  |  |  |  |  |  |  |  |  |  |  |  |  |  |  |  |  |  |  |  |  |  |  |
| Anxiety, stress & depression control |  | ✓ |  |  |  | ✓ |  |  |  |  |  |  |  |  | ✓ |  | ✓ |  | ✓ |  |  |  |  |  |  |  |  |  |  |  |  |
| Unanxiety: Stress relief | ✓ |  |  |  | ✓ |  |  |  |  |  |  |  |  |  |  |  |  |  |  |  |  |  |  |  |  |  |  |  |  |  |  |
| Way To De-Stress | ✓ | ✓ |  |  |  |  |  |  |  |  |  |  |  |  |  |  |  |  |  |  |  |  |  |  |  |  |  |  |  |  |  |
| Welltory: Heart Rate Monitor | ✓ | ✓ | ✓ |  |  |  |  | ✓ |  |  |  |  |  |  | ✓ |  | ✓ |  |  |  |  |  |  |  |  |  |  |  | ✓ |  | ✓ |
| White Noise : Calm, stress reduction, relaxing. | ✓ |  |  |  |  |  |  |  |  |  |  |  | ✓ |  |  |  |  |  |  |  |  |  |  |  |  |  |  |  |  |  |  |
| White Noise Sleep Sounds: Rain, Nature, and more | ✓ |  |  |  |  |  |  |  |  |  |  |  |  |  | ✓ | ✓ |  |  |  |  |  |  |  |  |  |  |  |  |  |  |  |
| Wim Hof Method | ✓ | ✓ |  | ✓ | ✓ |  |  |  |  |  |  |  |  |  | ✓ | ✓ | ✓ |  |  |  |  |  |  |  |  |  |  |  |  |  |  |
| Wysa: stress, depression & anxiety therapy chatbot | ✓ | ✓ |  | ✓ |  |  |  |  | ✓ |  |  |  |  |  |  | ✓ | ✓ |  |  | ✓ |  |  |  |  |  |  |  |  |  |  | ✓ |
